# Biosynthesis of H_2_S and Siderophores Targeting Gram‐Negative Bacterial Resistance to Reactive Oxygen Species

**DOI:** 10.1002/advs.202505748

**Published:** 2025-09-15

**Authors:** Congyang Mao, Wanyu Jin, Yiming Xiang, Yizhou Zhu, Jun Wu, Xiangmei Liu, Shuilin Wu, Wei Qiao, Kenneth M. C. Cheung, Kelvin Wai Kwok Yeung

**Affiliations:** ^1^ Department of Orthopaedics & Traumatology Li Ka Shing Faculty of Medicine The University of Hong Kong Pokfulam Hong Kong 999077 China; ^2^ Shenzhen Key Laboratory for Innovative Technology in Orthopaedic Trauma Department of Orthopaedics and Traumatology The University of Hong Kong‐Shenzhen Hospital Shenzhen 518053 China; ^3^ School of Life Science and Health Engineering Hebei University of Technology Xiping Avenue 5340, Beichen Tianjin 300401 China; ^4^ School of Materials Science and Engineering Peking University Beijing 100871 China

**Keywords:** Gram‐negative bacteria, hydrogen sulfide, outer membrane biogenesis, pyoverdine siderophores, ROS resistance, ROS scavenging

## Abstract

Reactive oxygen species (ROS) are a promising alternative bactericide. However, it is questioned that bacteria can potentially develop resistance to ROS, similar to their resistance against antibiotics and silver. Herein, it is reported that Gram‐negative bacteria, including *Pseudomonas aeruginosa*, *Escherichia coli*, and *Klebsiella pneumoniae*, develop resistance to ROS after six repeated exposures. Notably, ROS minimum inhibitory concentration of *Pseudomonas aeruginosa* significantly increases to 256‐fold after ten passages. The resistance mechanism predominantly originates from the intensified biosynthesis of the highly reductive hydrogen sulfide (H_2_S) and pyoverdine (PVD) siderophores, effectively neutralizing ROS. Simultaneously, PVD transports Fe^3+^ from the extracellular space into the bacteria, releasing H_2_S bound to Fe^3+^ and enhancing ROS scavenging. Additionally, the enhanced outer membrane (OM) biogenesis establishes a robust OM barrier, impeding ROS penetration. The acquired resistance to ROS can be significantly reduced by incorporating additional Fe^3+^ into the culture medium or disrupting the H_2_S biosynthetic gene. These observations suggest that careful consideration is required when utilizing ROS against Gram‐negative bacteria. It is anticipated that understanding this resistance mechanism can inform the development of future antimicrobial agents, particularly for Gram‐negative bacteria.

## Graphical abstract

Discover how Gram‐negative bacteria develop resistance to reactive oxygen species, and how scientists aim to overcome this challenge.

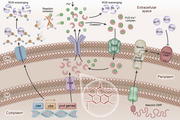

## 1 Introduction

The global public health crisis caused by bacterial resistance to antibiotics necessitates investigating alternative approaches to address infections.^[^ ^1^, ^2^, ^3^ ^]^ Reactive oxygen species (ROS) are chemically reactive oxygen‐containing molecules, produced as byproducts of regular cellular metabolism or in response to environmental factors.^[^ ^4^, ^5^ ^]^ Despite potential adverse effects on cellular components, ROS have been recognized for their extraordinary antimicrobial properties by damaging cellular macromolecules, such as DNA, proteins, and lipids, ultimately leading to the death of bacterial cells.^[^ ^6^, ^7^ ^]^ With the increasing threat of antibiotic resistance, ROS could potentially serve as an alternative or adjunct to conventional antibiotics for eradicating bacteria, including multidrug‐resistant strains.^[^ ^8^, ^9^, ^10^ ^]^ The utilization of ROS in medical and biotechnological applications has rapidly expanded in recent years, encompassing a wide range of areas such as antimicrobial coatings and wound dressings, targeted drug delivery systems, and cancer therapies.^[^ ^11^, ^12^, ^13^ ^]^ At present, it is premature to ascertain whether ROS could be further employed in clinical practice to boost the effectiveness of antibiotics or completely replace them in treating local and systemic infections. However, as researchers continue to explore the capabilities and limitations of ROS, comprehending the possible risks associated with their use, such as the emergence of bacterial resistance, becomes increasingly imperative.

Numerous antibiotics, such as fluoroquinolone, *β*‐lactams, and aminoglycosides, exert their bactericidal effects by augmenting ROS production in bacteria.^[^ ^14^ ^]^ Oxidative stress arises when an imbalance exists between ROS production and the capacity of an organism to detoxify these reactive molecules, causing damage to the bacteria.^[^ ^15^, ^16^ ^]^ It is noteworthy that the primary bactericidal mechanism of ROS involves the induction of oxidative stress in bacterial cells, leading to damage and eventual cell death.^[^ ^17^ ^]^ In response to ROS‐induced damage, bacterial cells have developed antioxidant defense mechanisms, such as catalase and superoxide dismutase enzymes, to neutralize ROS and maintain cellular redox balance.^[^ ^18^, ^19^ ^]^ In addition, it has been observed that Gram‐negative bacteria can surprisingly develop resistance to silver,^[^ ^20^, ^21^ ^]^ which is widely recognized for its potent antibacterial properties. However, to date, it seems that study rarely concerns the development of bacterial resistance to ROS attack.

In this study, we aimed to investigate the potential of bacteria to develop resistance to ROS, similar to the resistance observed in antibiotics and silver.^[^ ^20^, ^21^, ^22^ ^]^ Utilizing experimental evolution, we identified a unique mechanism by which Gram‐negative bacteria counteract ROS attacks after prolonged and repeated exposures. To simulate the process of bacterial resistance development, we repeatedly evolved both Gram‐positive and Gram‐negative bacteria in sub‐inhibitory concentrations of ROS, allowing us to determine whether distinct ROS resistance mechanisms evolved in different bacterial types. Our observations revealed that Gram‐negative bacteria rapidly acquire resistance to ROS throughout the evolutionary process, while Gram‐positive bacteria seldom develop such resistance relatively. Further analysis of the ROS resistance mechanisms in Gram‐negative bacteria led to the discovery of a novel triple‐synergistic defense barrier, consisting of hydrogen sulfide (H_2_S), siderophores (pyoverdine), and an outer membrane barrier. Our findings collectively shed light on the mechanisms of ROS resistance and provide strategies to overcome this resistance through advanced technology.

## 2 Results

### 2.1 Development of Bacterial Resistance to ROS Attack

For the purposes of this investigation, the most prevalent and readily available ROS, hydrogen peroxide (H_2_O_2_), was employed to induce resistance, which was assessed through the determination of minimum inhibitory concentration (MIC). With the H_2_O_2_ in hand, ten successive bacterial cultivation stages were conducted for each bacterial strain in the presence of different H_2_O_2_ concentrations (Figure S1, Supporting Information). In the 96‐well plates, the H_2_O_2_ concentration was sequentially diminished by half from column 1 – 12, where the concentration of 1F‐H was half of 12A‐C and then sequentially reduced by half; this procedure was replicated thrice for each concentration. After 24 h of coculture, the H_2_O_2_ concentration in the clear wells immediately adjacent to the turbid wells was documented as MIC (Figure S2, Supporting Information). The treated wild‐type [WT, passage 1 (P1)] bacteria that survived in these turbid wells with the highest H_2_O_2_ concentration were collected as the subsequent bacterial passage and subsequently rechallenged using an identical methodology until passage 10 (P10). The H_2_O_2_ MICs were ultimately ascertained after each passage.

**Figure 1:**
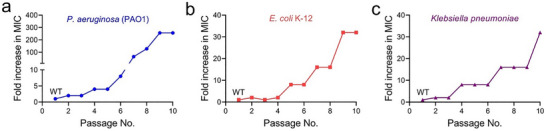
Evolution of ROS resistance in various bacterial strains. a–c) The development of ROS resistance in Gram‐negative bacteria, including a) *P. aeruginosa* (PAO1), b) *E. coli* K‐12, and c) *Klebsiella pneumoniae*, is depicted in response to H_2_O_2_. This is represented by a fold increase in MIC versus the passage number. All experiments were performed in triplicate, with a sample size of three (n = 3).

Employing this approach*, Pseudomonas aeruginosa* (*P. aeruginosa*, PAO1) was the first bacterium to display appreciable resistance to the ROS. An eightfold increase in MIC was observed for this strain after six repeated passages with H_2_O_2_ (**Figure** **1**a), suggesting the emergence of H_2_O_2_ resistance.^[^ ^23^ ^]^ Notably, the strain experienced a 256‐fold MIC increase (from 18.3 to 4688 µg mL^−1^, Table S1, Supporting Information) upon repeated exposure to H_2_O_2_ within ten passages, signifying the extreme susceptibility of *P. aeruginosa* PAO1 to develop ROS resistance. In a similar manner, *Escherichia coli* (*E. coli*, K‐12) and *Klebsiella pneumoniae* (ATCC10031) also developed resistance to the H_2_O_2_ after ten repeated passages (Figure 1b,c; Table S1, Figure S2, and S3, Supporting Information). These results unequivocally revealed that all Gram‐negative bacteria with ROS resistance displayed significantly elevated MIC values in comparison to their ROS‐susceptible progenitor strains, demonstrating that all examined Gram‐negative bacterial strains developed resistance to the H_2_O_2_ following repeated exposure.

### 2.2 H_2_S Biogenesis‐Mediated Defense System

**Figure 2:**
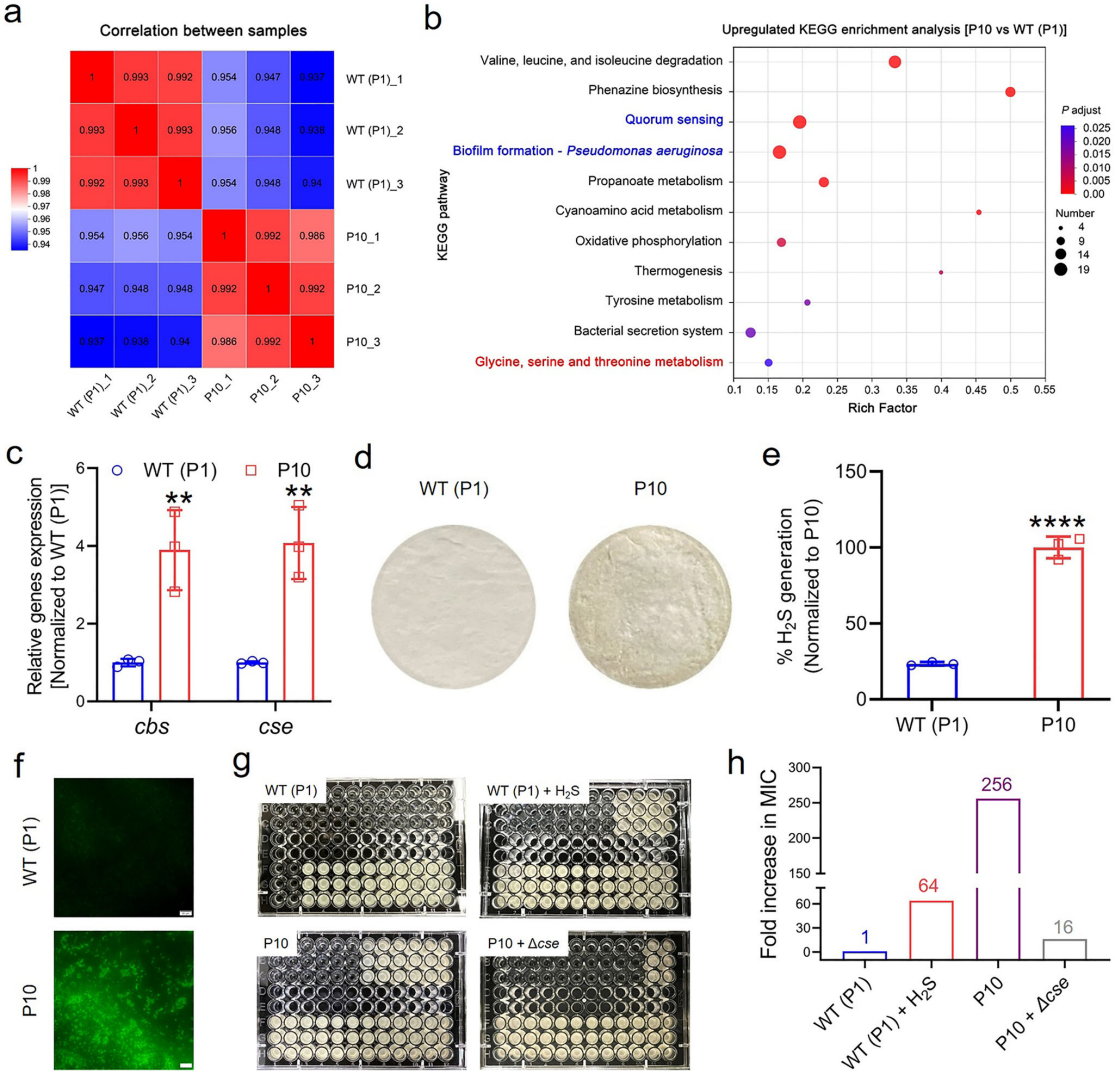
H_2_S generation‐mediated defense system against ROS. a) Correlation analysis between the WT (P1) and P10 bacterial strains. Differently colored squares represent the varying degrees of correlation between the two samples, with a larger correlation coefficient indicating a higher correlation. b) Bubble map illustrating the most significant enrichment in the upregulated KEGG terms in P10 bacterial strain compared to the WT (P1) bacterial strain. (Fisher exact test was employed, adjusted *P* <0.05) c) Relative expression of *cbs* and *cse* genes in the WT (P1) and P10 bacterial strains. Data were obtained from independent samples (n = 3). Error bars represent the mean ± standard deviation, with significance level indicated as **p <0.01. A two‐sample Student *t*‐test was utilized for the statistical analysis. d–f) Quantitation of H_2_S production from the WT (P1) and P10 bacterial strains. d) Representative lead acetate‐soaked filter papers exhibit a brown stain of lead sulfide, resulting from the reaction with gaseous H_2_S. e) Relative H_2_S production and f) the corresponding fluorescent images in the WT (P1) and P10 bacterial strains treated with the WSP5 fluorescent H_2_S probe. Data were obtained from independent samples (n = 3). Error bars represent the mean ± standard deviation, with significance level indicated as ****p <0.0001. A two‐sample Student *t*‐test was utilized for the statistical analysis. g) Photographs of the 96‐well plates containing WT (P1) bacteria (Top left), WT (P1) bacteria and the H_2_S donor NaHS (0.4 mm, top right), P10 bacteria (Bottom left), and P10 bacteria with *cse* gene knockout (Δ*cse*, bottom right) after cocultivation with H_2_O_2_ (Concentration decreasing 23 times in half from left to right, top to bottom) at 37 °C for 24 h. h) The corresponding fold increase in MIC of each treatment. All experiments were performed in triplicate, with a sample size of three (n = 3).

The fold increase in MICs observed in our investigation implied that *P. aeruginosa* PAO1 bacterial resistance to ROS developed more readily in comparison to other Gram‐negative bacterial strains. We then employed RNA sequencing to explore the underlying resistant mechanisms in ROS‐resistant *P. aeruginosa* PAO1. Initially, a heat map was constructed based on the Pearson correlation coefficient calculation between the pairings of WT (P1) and P10 (**Figure** **2**a), which is a prevalent technique employed to exhibit the extent of linear correlation between two datasets.^[^ ^24^ ^]^ All the calculated correlation coefficients fell within the acceptable ranges (|*r*| > 0.6).

Subsequently, we utilized the Kyoto Encyclopedia of Genes and Genomes (KEGG), a prominent database resource for bioinformatics analysis, to explicate the intricate functionalities and utilities inherent within biological systems, including cellular, organismal, and ecological levels.^[^ ^25^ ^]^ We observed an augmented metabolism of glycine, serine, and threonine in P10 ROS‐resistant bacteria compared to WT (P1) ROS‐susceptible bacterial strain (Figure 2b). Further, we executed a KEGG functional enrichment analysis on the genes to clarify their involvement in KEGG signaling pathways.^[^ ^26^ ^]^ Notably, cystathionine γ‐lyase (CSE) and cystathionine β‐synthase (CBS) participated in the glycine, serine, and threonine metabolism, with the corresponding *cse* and *cbs* genes displaying significant upregulation in P10 ROS‐resistant bacterial strain (Figure 2c; Figure S4, Supporting Information). Another crucial role of CSE and CBS in bacteria involves the catalysis of the substrate cysteine to yield hydrogen sulfide (H_2_S),^[^ ^27^, ^28^, ^29^ ^]^ which protects the bacteria from antibiotic‐induced oxidative stress.^[^ ^30^, ^31^ ^]^ We detected a marked enhancement in cysteine metabolism in the P10 ROS‐resistant strain, predominantly regulated by *cse* and *cbs* (Figure S4, Supporting Information). Consequently, we hypothesized that the upregulation of *cse* and *cbs* bolstered H_2_S production in the P10 ROS‐resistant strain, thereby shielding bacteria from ROS‐induced oxidative stress and facilitating the emergence of bacterial resistance to ROS.

To detect H_2_S production, we initially employed the classic lead acetate reactivity test,^[^ ^32^ ^]^ in which a filter paper saturated in a 2% lead acetate solution is positioned over a well plate containing bacterial suspension. The H_2_S produced by the bacteria subsequently reacts with lead acetate, yielding a brownish precipitate of lead sulfide (Figure S5, Supporting Information). The findings demonstrated that the P10 ROS‐resistant strain produced a higher amount of H_2_S following 24 h of cultivation (Figure 2d,e). The H_2_S–nucleophilic substitution‐cyclization–based fluorescent probe analysis further substantiated the significant enhancement of H_2_S production in the P10 strain (Figure 2f).^[^ ^33^ ^]^ Consequently, we artificially supplemented H_2_S by introducing the H_2_S donor NaHS into the suspension of ROS‐susceptible WT strain, which strikingly acquired resistance, resulting in a 64‐fold increase in MIC of H_2_O_2_ (Figure 2g,h). Nevertheless, this increase was markedly lower than the 256‐fold enhancement observed in P10 ROS‐resistant strain. On the other hand, CSE has been identified as the primary generator of H_2_S.^[^ ^34^ ^]^ Thus, we endeavored to block the source of bacterial H_2_S by knocking out the *cse* gene in P10 ROS‐resistant strain, leading to a reduction in the increased MIC from 256‐fold to 16‐fold (Figure 2g,h). Although the resistance was diminished, the P10 + Δ*cse* strain still retained substantial ROS resistance. Collectively, these results imply the existence of supplementary resistant mechanisms within the P10 ROS‐resistant strain.

### 2.3 The Biogenesis of Siderophores and Their Mediated Synergistic ROS Resistance

**Figure 3:**
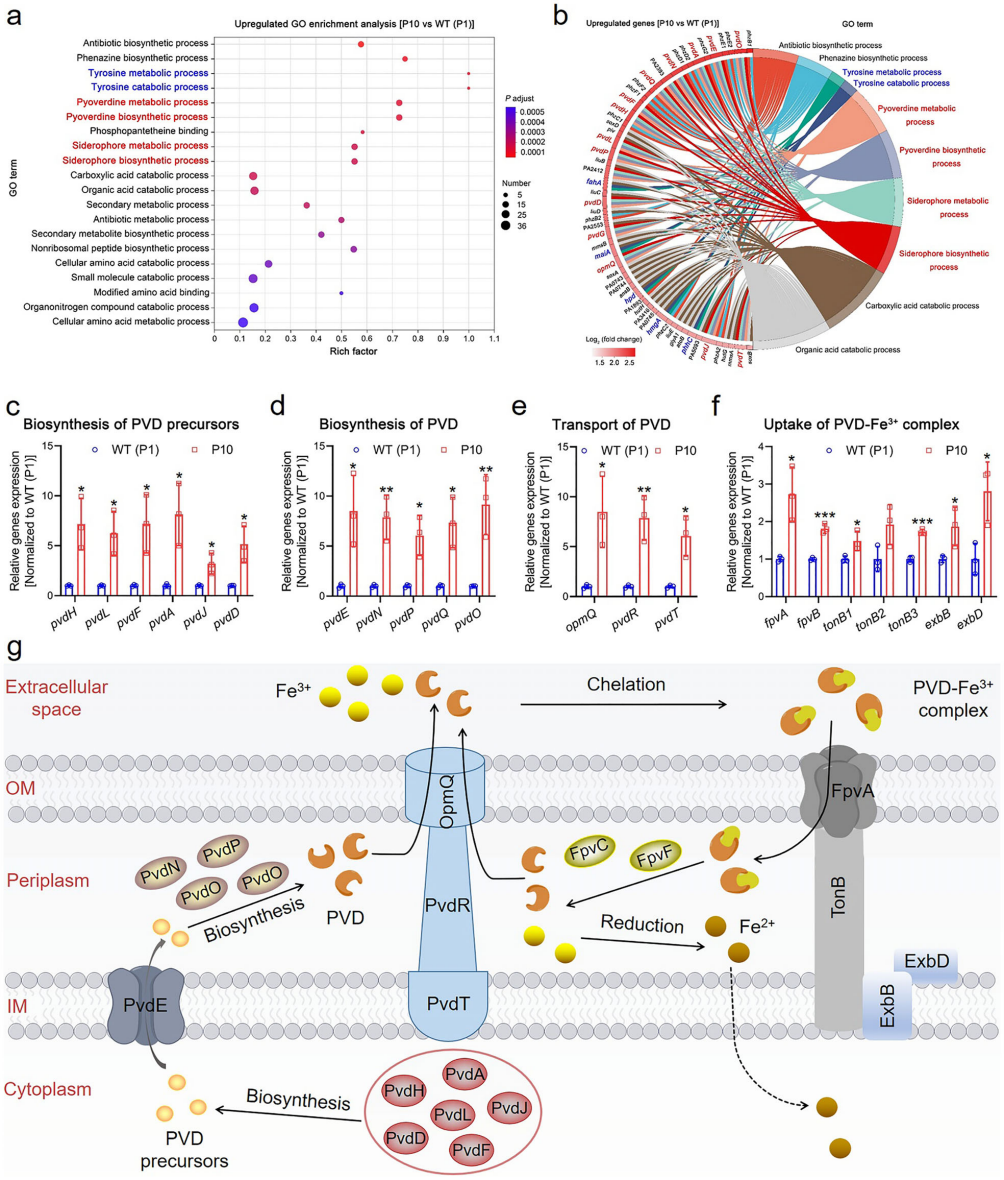
Augmented biosynthesis of PVD and the uptake of Fe^3+^. a,b) a)Bubble map and b) chord plot illustrating the most significant enrichment in the upregulated GO terms in P10 bacterial strain compared to the WT (P1) bacterial strain. (Fisher exact test was employed, adjusted *P* <0.05) c–f) Relative expression of genes associated with biosynthesis of c) PVD precursors, d) biosynthesis of PVD, e) transport of PVD, and f) uptake of PVD‐Fe^3+^ complex in the WT (P1) and P10 bacterial strains. Data were obtained from independent samples (n = 3). Error bars represent the mean ± standard deviation, with significance levels indicated as *p <0.05, **p <0.01, and ***p <0.001. A two‐sample Student *t*‐test was utilized for the statistical analysis. g) Schematic diagram of biosynthesis of PVD and the uptake of Fe^3+^ in *P. aeruginosa*. The enzymes (PvdH, PvdL, PvdF, PvdA, PvdJ, PvdD, PvdN, PvdP, PvdQ, and PvdO) modulate the biosynthesis of PVD from the precursor to the mature form. Subsequently, PVD is released into the extracellular space via the efflux pump PvdRT‐OpmQ. *P. aeruginosa* bacteria further internalize the PVD‐Fe^3+^ complex for Fe^3+^ uptake following extracellular Fe^3+^ chelation. The dissociated Fe^3+^ is reduced to Fe^2+^ within the periplasm and transported to the cytoplasm, while the separated PVD is recycled back to the extracellular space.

To further explore potential ROS resistance mechanisms, we carried out Gene Ontology (GO) enrichment analysis on the RNA sequencing results. The results demonstrated a significant enhancement of pyoverdine (PVD) metabolic and biosynthesis processes in the P10 ROS‐resistant strain (**Figure** **3**a). We subsequently performed GO functional significance enrichment analysis on upregulated genes to further examine PVD biosynthesis and metabolism. We discovered that the metabolism of PVD and siderophores was governed by several common genes (Figure 3b), which is due to PVD acting as a siderophore for iron scavenging.^[^ ^35^ ^]^ Moreover, we identified that the biosynthetic regulatory genes for PVD and its precursors were upregulated (Figure 3c,d), along with the regulatory genes involved in PVD transport and eventual participation in iron scavenging (Figure 3e,f).

In general, PVD biosynthesis initiates in the cytoplasm, terminates in the periplasm, and is ultimately secreted into the extracellular space (Figure 3g). Given that PVD is a quorum sensing molecule, we observed a substantial enhancement of the quorum sensing pathway in KEGG enrichment analysis (Figure 2b). The genes accountable for PVD are located at the *pvd* locus, and enzymes PvdH, PvdL, PvdF, PvdA, PvdJ, and PvdD regulate the biosynthesis of PVD precursors in the cytoplasm, which are subsequently transported across the inner membrane (IM) to the periplasm by the PvdE ATP‐binding cassette (ABC)‐transporter. Ultimately, PVD biosynthesis in the periplasm is regulated by enzymes PvdN, PvdP, PvdQ, and PvdO, and PVD is released into the extracellular space via the efflux pump PvdRT‐OpmQ. Upon extracellular Fe^3+^ chelation, bacteria internalize the PVD‐Fe^3+^ complex for Fe^3+^ uptake. This complex is recognized by FpvA in concert with TonB and its partners ExbB and ExbD. This pump then transports the complex across the outer membrane (OM) to the periplasm, where enzymes FpvC and FpvF catalyze the separation. The dissociated Fe^3+^ is reduced to Fe^2+^ in the periplasm and transported to the cytoplasm, while the separated PVD is recycled back to the extracellular space.

Overall, the phenolic hydroxyl structure of PVD endows it with potent antioxidative capacity (Figure S6, Supporting Information), and its abundant distribution in the extracellular space can protect the bacteria from ROS damage in the microenvironment, thereby gradually developing ROS resistance. More importantly, the presence of oxidative Fe^3+^ in the extracellular space may also interact with and deplete H_2_S, resulting in a reduced availability of H_2_S for ROS scavenging, which is detrimental to bacterial protection. In P10 ROS‐resistant strain, however, the enhanced biosynthesis of PVD facilitates the transportation of substantial amounts of Fe^3+^ from the extracellular space into the bacteria, resulting in a considerable reduction of Fe^3+^ available in the extracellular space for the consumption of H_2_S. This allows for an increased utilization of H_2_S for ROS scavenging, which is beneficial for the development of bacterial ROS resistance.

Furthermore, the biosynthesis of another pyochelin (PCH) siderophore and the uptake of Fe^3+^ by PCH were also substantially enhanced in the P10 ROS‐resistant strain (Figure S7a,b, Supporting Information). This further decreased extracellular Fe^3+^ levels, consequently enhancing the antioxidative capacity of the P10 bacteria. Notably, both PVD and PCH siderophores are distinctive metabolic products of *P. aeruginosa*,^[^ ^36^ ^]^ implying that *P. aeruginosa* exhibits a specialized siderophores‐mediated defense mechanism against ROS. As a result, *P. aeruginosa* demonstrated a higher propensity for developing ROS resistance when compared to other Gram‐negative bacteria. Additionally, we detected a marked intensification in the biosynthesis of a phenazine antibiotic (Figure 3a,b). This phenomenon can be ascribed to the considerable translocation of Fe^3+^ into the bacterium, leading to the synthesis of large quantities of phenazine in iron‐deficient media, thereby facilitating bacterial iron metabolism.^[^ ^37^ ^]^

### 2.4 Outer Membrane Barrier Against ROS and Other Potential ROS‐Resistant Pathways

**Figure 4:**
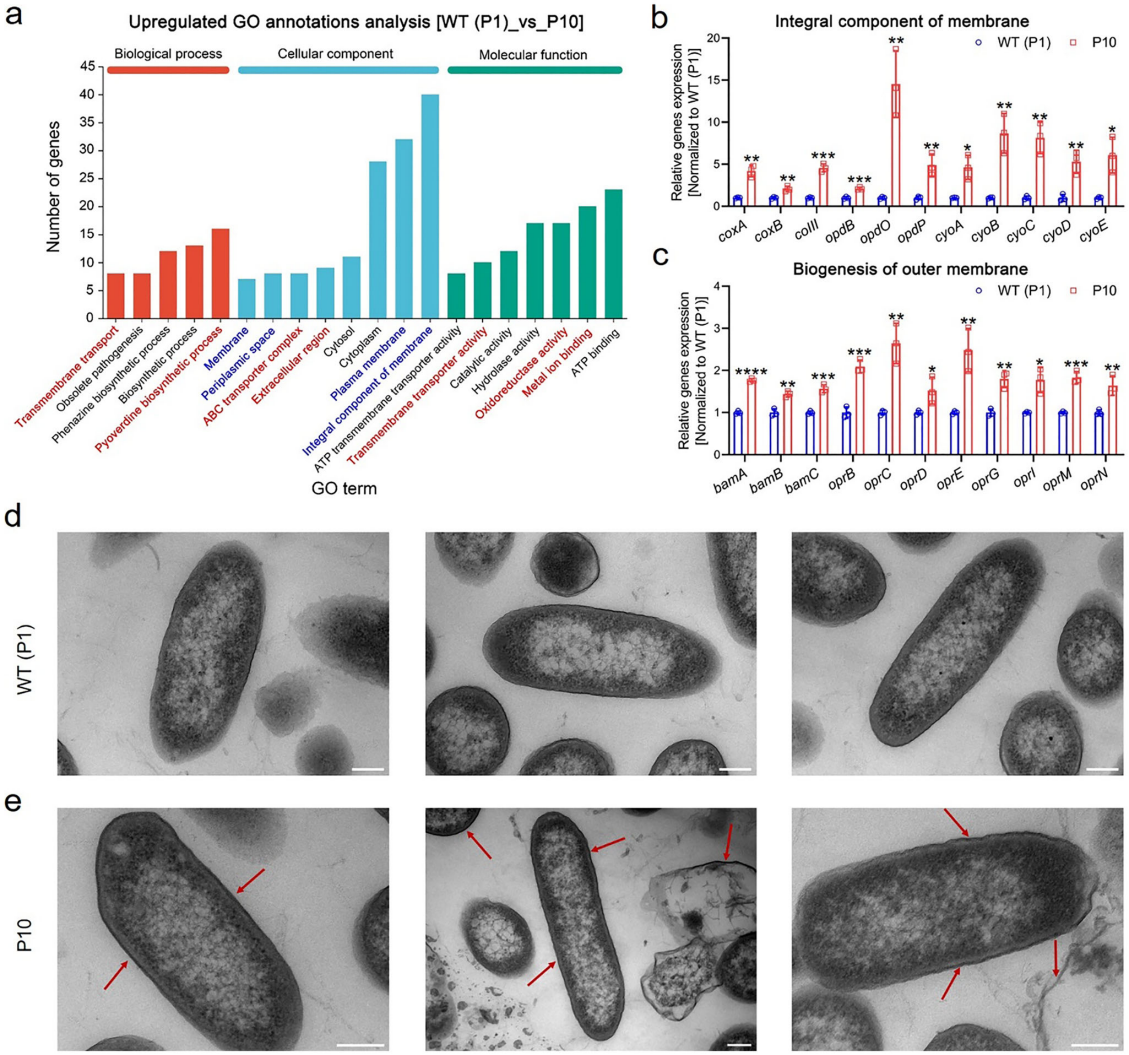
BamA protein‐mediated outer membrane barrier against ROS. a) Analysis of upregulated GO annotations (biological process, cellular component, and molecular function) in the P10 bacterial strain compared to the WT (P1) bacterial strain. Data were obtained from independent samples (n = 3). b,c) Relative expression of genes related to b) integral component of the membrane and c) biogenesis of outer membrane in the WT (P1) and P10 bacterial strains. Data were obtained from independent samples (n = 3). Error bars represent the mean ± standard deviation, with significance levels indicated as *p <0.05, **p <0.01, and ***p <0.001. A two‐sample Student *t*‐test was utilized for the statistical analysis. d,e) Biological transmission electron microscopy images of the cross‐sectional structure of d) the WT (P1) and e) P10 bacterial membrane. Red arrows highlight the robust, dense, and compact bacterial cell membranes.

Finally, we performed a GO annotations analysis on the upregulated genes to investigate their involvement in the biological process, cellular component, and molecular function.^[^ ^38^ ^]^ Our results revealed that transmembrane transport, pyoverdine biosynthetic process, ABC transporter complex, extracellular region, oxidoreductase activity, and metal ion binding—all involved in siderophores and H_2_S biogenesis processes—were significantly augmented (**Figure** **4**a). This further substantiated the synergistic defense mechanism against ROS by siderophores and H_2_S production. Additionally, among the cellular components exhibiting increased expression, we unexpectedly discovered that the primary upregulated genes were predominantly enriched in the plasma membrane and integral components of the membrane. Moreover, the genes modulating the integral component of the membrane and OM biogenesis were also markedly upregulated (Figure 4b,c). We noted that the *bamA* gene was substantially enhanced, which encodes the BamA protein. The BamA protein constitutes the core component of a protein complex known as the β‐barrel assembly machine (BAM), through which outer membrane proteins (OMPs) are captured, inserted, and folded onto the OM.^[^ ^39^ ^]^ Consequently, the significant upregulation of the bamA gene results in the insertion and folding of more OMPs onto OM, establishing a dense and robust OM barrier that prevents the permeation of ROS into the bacteria, thereby further protecting the bacteria from oxidative stress‐induced damage. Consequently, we first examined bacterial morphology using scanning electron microscopy (SEM). The WT (P1) ROS‐susceptible and P10 ROS‐resistant strains exhibited no significant morphological differences under SEM observation (Figure S8, Supporting Information). We subsequently examined the cross‐sectional structure of the bacterial membrane using biological transmission electron microscopy. The membrane of the P10 ROS‐resistant bacteria was noticeably denser and more robust than that of the WT (P1) ROS‐susceptible bacteria (Figure 4d,e, denoted by the red arrows), further substantiating the ROS resistance induced by BamA protein‐mediated OM regulation.

We also noted an enhancement in the metabolic and catabolic processes of tyrosine (Figure 3a,b), which is an aromatic amino acid.^[^ ^40^ ^]^ A critical metabolic pathway for tyrosine involves its conversion to homogentisic acid under the catalysis of tyrosine aminotransferase,^[^ ^41^ ^]^ and the gene encoding aromatic amino acid aminotransferase, *phhc*, was also significantly upregulated (Figure S9, Supporting Information). Consequently, we inferred that the abundant production of homogentisic acid from tyrosine, catalyzed by aromatic amino acid aminotransferase, confers potent antioxidative capacity due to its phenolic hydroxyl structure (Figure S9b, Supporting Information), thereby facilitating the scavenging of ROS. Lastly, the regulatory genes related to biofilm biosynthesis and formation were also markedly upregulated (Figure S9c,d, Supporting Information). And KEGG enrichment analysis disclosed a significant enhancement in the formation of *P. aeruginosa* biofilms (Figure 2b), which is governed by the intensified quorum sensing regulation.^[^ ^42^, ^43^ ^]^ The crystal violet staining was then used to assess biofilm formation capacity of different strains. Following 48 h of incubation, the P10 strain exhibited denser crystalline violet deposits and a higher absorbance at 590 nm (Figure S10, Supporting Information), demonstrating its enhanced biofilm‐forming capability. However, adaptive resistance typically incurs a fitness cost. Consequently, we characterized the growth kinetics of the strains. We observed a reduced planktonic growth rate in the P10 strain (Figure S11, Supporting Information), potentially attributable to the reallocation of energy resources toward biofilm development. Consistent with this phenotype, the P10 strain displayed significant upregulation of the *relA* gene (Figure S12, Supporting Information). This gene orchestrates the bacterial stringent response, which confers tolerance to ROS while simultaneously inhibiting ribosomal biosynthesis, thereby slowing growth. Additionally, significant upregulation of the phz operon was observed in the P10 strain (Figure S13, Supporting Information), which encodes the pyocyanin virulence factor implicated in *P. aeruginosa* biofilm formation. The protective effect of the biofilm further increases the tolerance of bacteria to ROS assaults.

To further validate the detoxification capacity of the P10 bacterial strain toward ROS, its tolerance to general ROS was first assessed using DCFH‐DA. Fluorescence intensity serves as a direct indicator of intracellular oxidative stress levels. We observed that P10 ROS‐resistant strain exhibited significantly weaker fluorescence intensity following ROS treatment compared to WT (P1) ROS‐susceptible bacteria (Figure S14, Supporting Information), indicating superior antioxidant capability of P10 strain. To confirm that the P10 ROS‐resistant bacterial strain also exhibits resistance to other ROS, we treated bacteria with more potent singlet oxygen (^1^O_2_) and hydroxyl radical (•OH). When P10 bacteria were mixed with 1,3‐diphenylisobenzofuran (DPBF) and subjected to the ^1^O_2_‐generating photosensitizer Chlorin e6, they showed the biggest in absorbance (OD value) (Figure S15, Supporting Information), suggesting enhanced scavenging of ^1^O_2_ by P10. Quantitative analysis revealed a ^1^O_2_ scavenging rate of 41.24% for the P10 strain. Subsequently, after mixing P10 bacteria with a •OH‐generating system (FeSO_4_‐H_2_O_2_) for 10 min, the lowest characteristic peak intensity for •OH was detected (Figure S16, Supporting Information). These results collectively demonstrated that the P10 strain possessed a robust detoxification capacity against ROS. Moreover, both the ^1^O_2_ and •OH eradicated more than 95% of the WT (P1) ROS‐susceptible bacteria within 10 min, while only ≈40% of the P10 ROS‐resistant strain was eliminated (Figure S17, Supporting Information), further demonstrating the enhanced tolerance of P10 strain to ROS. Intriguingly, a parallel set of experiments involving the Gram‐positive bacterium, *Staphylococcus aureus* (*S. aureus*, USA300), revealed slight and negligible fluctuations in the MIC of ROS (Figures S18 and S19 and Table S2, Supporting Information), indicating that no obvious *S. aureus* bacterial resistance to ROS emerged under the identical treatment conditions. Consequently, the induced bacterial resistance to ROS appears to Gram‐negative bacterial strains exclusively.

### 2.5 Regulation of ROS Resistance

**Figure 5:**
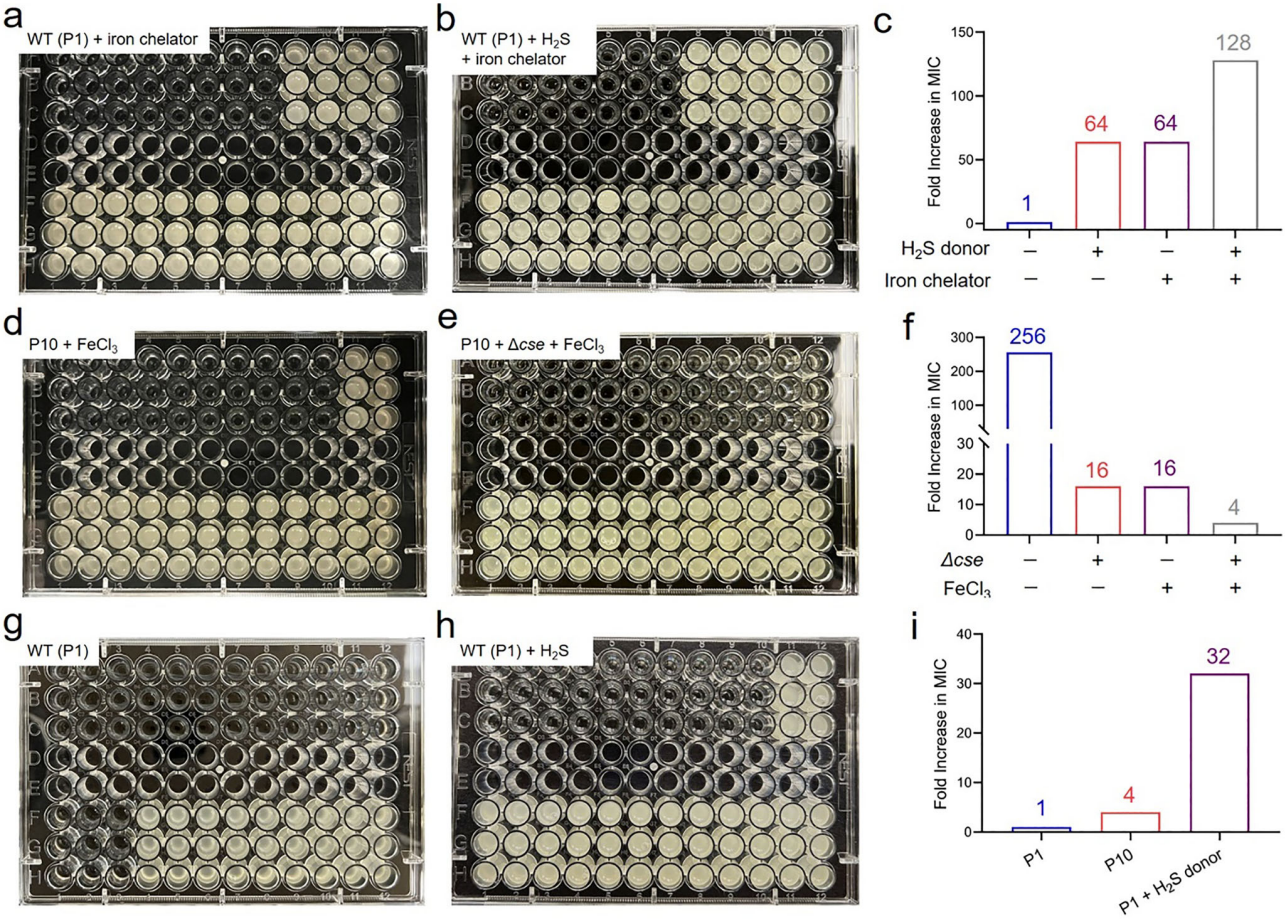
H_2_S generation and PVD‐mediated synergetic defense system against ROS. a–c) Photographs of the 96‐well plates containing WT (P1) *P. aeruginosa* bacteria with a) iron chelator, b) the H_2_S donor NaHS (0.4 mm) and iron chelator after cocultivation with H_2_O_2_ (Concentration decreasing 23 times in half from left to right, top to bottom) at 37 °C for 24 h and c) corresponding fold increase in MIC of each treatment. d–f) Photographs of the 96‐well plates containing P10 *P. aeruginosa* bacteria with d) Fe^3+^ supplementation, e) P10 *P. aeruginosa* bacteria with *cse* gene knockout (Δ*cse*) and f) Fe^3+^ supplementation after cocultivation with H_2_O_2_ (Concentration decreasing 23 times in half from left to right, top to bottom) at 37 °C for 24 h and corresponding fold increase in MIC of each treatment. g–i) Photographs of the 96‐well plates containing g) WT (P1) *S. aureus* USA300 bacteria, h) WT (P1) *S. aureus* USA300 bacteria with H_2_S donor NaHS (0.4 mm) after cocultivation with H_2_O_2_ (Concentration decreasing 23 times in half from left to right, top to bottom) at 37 °C for 24 h and i) corresponding fold increase in MIC of each treatment. All experiments were performed in triplicate, with a sample size of three (n = 3).

To further validate the siderophores‐induced ROS resistance and its collaboration with the H_2_S‐mediated defensive barrier, we initially incorporated an iron chelator into the suspension of the ROS‐susceptible WT (P1) bacteria to eliminate Fe^3+^ from the culture medium, resulting in a 64‐fold increase in the MIC of H_2_O_2_ (**Figure** **5**a). Moreover, the bacterial resistance to ROS was further amplified after incorporating both the iron chelator and H_2_S donor NaHS into the medium (Figure 5b,c). Conversely, the resistance of the P10 ROS‐resistant bacteria significantly diminished upon the introduction of appropriate Fe^3+^ into the culture medium (Figure 5d), and this reduction in resistance was even more striking in the case of *cse* gene‐knockout bacterial cultures supplemented with Fe^3+^ (Figure 5e,f). These findings demonstrate that the augmented siderophores metabolism shields bacteria from ROS‐induced damage. And the robust defense systems, established by the synergistic action of siderophores metabolism and H_2_S biogenesis, are vital for bacterial resistance to ROS. Subsequently, we exaggeratedly introduced the H_2_S donor NaHS into the suspension of *S. aureus* USA300, and its resistance to H_2_O_2_ remarkably increased 32‐fold (Figure 5g–i), further confirming that H_2_S biogenesis constitutes one of the fundamental mechanisms for bacterial development of ROS resistance.

## 3 Discussion

**Figure 6:**
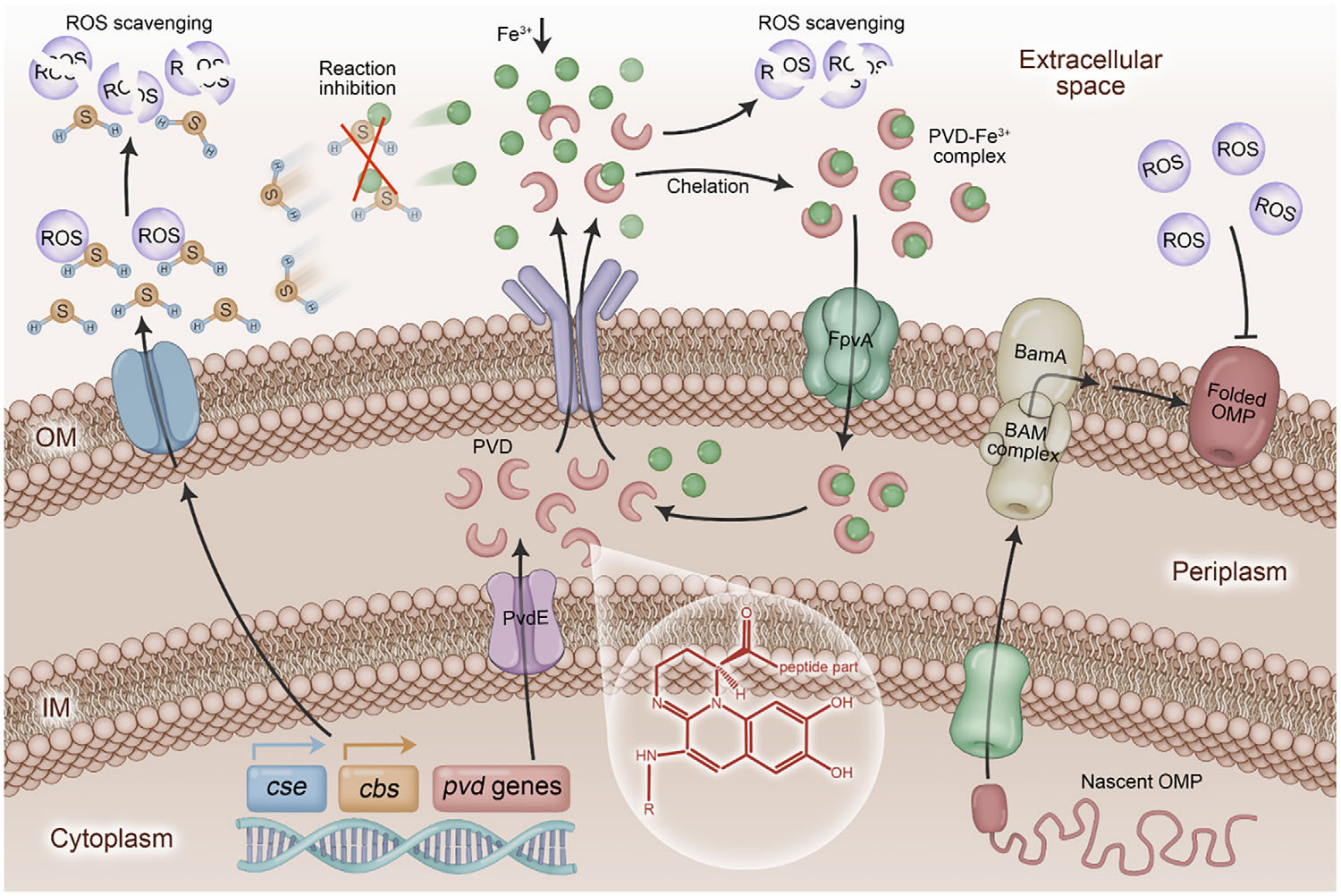
ROS resistance is primarily facilitated by a synergistic interplay of three defense barriers. The potent reducing property of H_2_S and the antioxidative capacity of the phenolic hydroxyl structures in PVD function to scavenge ROS. On the other hand, PVD transports a significant amount of Fe^3+^ into the bacteria, which in turn reactivates the H_2_S sequestered by Fe^3+^ for ROS scavenging. Both of these pathways contribute to the evolution of ROS resistance in Gram‐negative bacteria. Furthermore, the BamA protein‐mediated OM barrier impedes the penetration of ROS into the bacteria, thereby safeguarding Gram‐negative bacteria from ROS‐induced damage.

The findings from this study reveal distinct resistance responses in Gram‐negative bacteria following repeated exposure to subinhibitory concentrations of ROS, while Gram‐positive bacteria demonstrated less resistance relatively. This resistance is predominantly triggered by three synergistic barriers (**Figure** **6**). In Gram‐negative bacteria, the upregulation of *cse* and *cbs* genes mediates an increased production of H_2_S through cysteine metabolism, which facilitates ROS scavenging and alleviates bacterial oxidative stress due to the potent reducing properties of H_2_S. Furthermore, the enhanced siderophores metabolism in Gram‐negative bacteria, regulated by a series of upregulated *pvd* genes, results in a significant increase in extracellular PVD siderophores levels. The phenolic hydroxyl structure of PVD confers a robust antioxidative property, enabling it to scavenge extracellular ROS. Additionally, PVD chelates extracellular Fe^3+^ and transports it into the periplasm, consequently diminishing extracellular Fe^3+^ levels and limiting its involvement in H_2_S reactions, thereby enabling more H_2_S to scavenge ROS. The augmentation of folding in the OM of OMPs originating in the cytoplasm, mediated by BAM complex with BamA protein at its core, additionally contributes to a more robust, dense, and compact bacterial cell membrane. This, in turn, reinforces resistance against ROS penetration and subsequent damage. Owing to the unique presence of an OM in Gram‐negative bacteria,^[^ ^44^, ^45^ ^]^ they are more prone to develop resistance against ROS compared to Gram‐positive bacteria.

In a recent study, Galdino et al. made a surprising discovery that ancestral susceptible *P. aeruginosa* strains could survive high cephalosporin cefiderocol concentrations through production of the siderophore pyoverdine.^[^ ^46^ ^]^ Cefiderocol is particularly efficacious in treating carbapenem‐resistant *P. aeruginosa* infections in critically ill hospital or cystic fibrosis patients who possess limited therapeutic alternatives.^[^ ^47^ ^]^ Unfortunately, resistance to cefiderocol has also been documented in clinical isolates.^[^ ^48^ ^]^ Pyoverdine has the ability to chelate iron from cefiderocol, thereby diminishing its efficacy against *P. aeruginosa*. These findings further evidence that siderophores can serve unexpected protective cooperative roles in bacteria by the enhanced chelation of iron.

It is essential to mention that even without observed resistance in Gram‐positive bacteria, the potential for resistance to evolve over an extended period (e.g., beyond ten passages) cannot be ruled out. Hence, an in‐depth exploration is necessary to examine these potential scenarios. Additionally, we recognize that the unpredictable nature of evolutionary adaptation might lead to varying phenotypes or involve distinct genes,^[^ ^49^, ^50^ ^]^ resulting in multiple mechanisms and resistance levels. Consequently, examining and comprehending the impact of diverse ROS resistant mechanisms emerging in various populations is vital. The future study involving multiple populations could reveal further ROS resistant strategies. Furthermore, the factors such as exposure concentrations, durations, and the presence of other stressors will also impact the development of resistance.

Notably, the developed ROS resistance can be remarkably diminished by introducing extra Fe^3+^ ions into the culture medium or by knocking out the H_2_S metabolic *cse* gene. These findings highlight the essential mechanisms that may contribute to bacterial resistance against antibacterial agents, and can be further leveraged to identify key targets for developing innovative antimicrobial strategies. Furthermore, our findings recommend against the extensive use of ROS as a bactericidal agent (including photodynamic therapy, sonodynamic therapy, cold plasma, and immunoactivation) in treating Gram‐negative bacteria to avoid exacerbating the global resistance issue. Adopting this cautious strategy may aid in postponing or even averting the emergence of future ROS‐resistant superbugs, which would present an increased threat to human health and well‐being.

## 4 Conclusion

In summary, our research has demonstrated that Gram‐negative bacteria can rapidly acquire resistance to the antibacterial property of ROS following prolonged and repeated exposures. This resistance is predominantly triggered by a cooperative defense barrier mediated by H_2_S and PVD siderophore. On one hand, the strong reducing characteristic of H_2_S and the antioxidative capacity of the phenolic hydroxyl structure in PVD serve to neutralize ROS, consequently mitigating the oxidative stress and antibacterial activity of ROS against Gram‐negative bacteria. On the other hand, the oxidative Fe^3+^ ions present in the culture medium sequester H_2_S, which compromises the effectiveness of H_2_S in scavenging ROS. However, the PVD transports a considerable amount of Fe^3+^ into the bacteria, which reactivates the H_2_S sequestered by Fe^3+^ for ROS scavenging and contributes to the gradual development of ROS resistance in Gram‐negative bacteria. The BamA protein‐mediated OM barrier further obstructs the penetration of ROS into the bacteria, thereby protecting Gram‐negative bacteria from ROS‐induced damage. Importantly, this developed ROS resistance can be significantly suppressed by introducing additional Fe^3+^ ions to the culture medium or through the knockout of the H_2_S metabolic *cse* gene. These findings clarify essential mechanisms that potentially drive bacterial resistance to antibacterial agents and can be further exploited for vital targets in the development of the innovative antimicrobial approaches. Moreover, our results advise researchers to exercise caution when employing ROS as a bactericidal agent in treating Gram‐negative bacteria, as this could contribute to delaying or even preventing the emergence of future ROS‐resistant superbugs that pose an increased threat to human health and well‐being.

## 5 Experimental Section

### Chemicals

Unless otherwise indicated, all chemicals were procured from Sigma–Aldrich (USA). Lead acetate basic, 1,3‐diphenylisobenzofuran (DPBF), 5,5‐dimethyl‐1‐pyrroline‐N‐oxide (DMPO), FeSO_4_, and Chlorin e6 were bought from Aladdin Regents Co., Ltd (Shanghai, China). WSP5 was purchased from GlpBio (USA).

### Bacterial Strains and Cultivation

The wild‐type *P. aeruginosa* (PAO1), *E. coli* (K‐12), *Klebsiella pneumoniae* (ATCC10031), and *S. aureus* (USA300) bacterial strains were obtained from BeNa Culture Colletion Co., Ltd (Beijing, China). *P. aeruginosa*, *E. coli*, and *S. aureus* bacterial strains were grown in Luria–Bertani (LB) at 37 °C, while *Klebsiella pneumoniae* bacterial strain was cultured overnight in nutrient broth (NB) at 37 °C. Following overnight culturing, the bacterial strains were reintroduced to a fresh medium and allowed to proliferate until the exponential phase was reached. Subsequently, a 1000‐fold dilution was performed using the corresponding broth, yielding ≈10^6^ colony‐forming units (CFU) per mL for further investigation. Stock solutions of each passage microorganisms were formulated in the respective broth, supplemented with 25% glycerol, and stored at ‐80 °C.

### Quantifying Resistance in Response to ROS

The initial antimicrobial efficacy of ROS (in the form of H_2_O_2_) was assessed using standard planktonic minimum inhibitory concentration (MIC) measurements. These experiments were conducted in triplicate using 96‐well plates. The 100 µL of H_2_O_2_ solutions were serially diluted in bacterial broth and inoculated with 100 µL of different bacterial suspensions (1 × 10^6^ CFU mL^−1^), resulting in a total well volume of 200 µL. The final tested concentrations of H_2_O_2_ were 15%, 7.5%, 3.75%… until the twenty‐third half dilution. The mixtures were incubated at 37 °C for 24 h in shake incubator.

To investigate the evolution of resistance, bacteria were repeatedly exposed to ROS for ten successive passages. A wild‐type (WT) bacterial parent strain was prepared and used to inoculate a serially diluted series of H_2_O_2_. The MIC was determined as the lowest concentration of antimicrobial agent that inhibited visible growth of the tested microorganisms after 24 h. The sub‐MIC cultures (the first three wells below the MIC) with surviving bacteria were collected and diluted for 100 000 times. The 20 µL of diluted bacterial solution was then subcultured on agar at 37 °C for 24 h. A single colony was used for inoculum preparation at a density of 1 × 10^6^ CFU mL^−1^ in the subsequent passage, where the bacteria were exposed to the second new series of diluted H_2_O_2_. The entire procedure was the same as the description above from the initial inoculation to the preparation of the new inoculum. The process was repeated for up to ten passages, the MICs at each passage was recorded.

The fold increase in the MIC (as described by Equation (1) was determined by dividing the MIC for the passage of interest *N* by the MIC of the WT susceptible bacteria:

(1) $$\mathrm{MIC}\;\mathrm{fold}\;\mathrm{chang}e_{\mathrm{experimental}}=\frac{\mathrm{MI}C_{\mathrm{experimental}\;\mathrm{passage}\;N}}{\mathrm{MI}C_{\mathrm{WT}}}$$

where “experimental” refers to each passage with antimicrobial exposure (e.g., passage 5  =  5 passages of antimicrobial exposure).

### RNA Sequencing Analysis

The experiment utilized the TruSeqTM Stranded Total RNA Library Prep Kit to construct libraries, replacing dTTP with dUTP in the dNTPs reagent during the synthesis of the second cDNA strand, resulting in the inclusion of A/U/C/G bases in the second cDNA strand. Before PCR amplification, the second cDNA strand was digested using uracil‐DNA glycosylase (UNG) enzyme, ensuring that the library contained only the first cDNA strand.

- Total RNA extraction: Total RNA was extracted from tissue samples, and the concentration and purity of the extracted RNA were assessed using a Nanodrop2000. RNA integrity was evaluated through agarose gel electrophoresis, and the RNA Integrity Number (RIN) was measured using an Agilent 2100. Each library construction required a total RNA amount of 2 µg, a concentration ≥ 100 ng µL^−1^, and an OD260/280 ratio between 1.8 and 2.2.
- rRNA removal: Unlike eukaryotic mRNA, prokaryotic mRNA does not have a polyA tail at the 3′ end, preventing the separation of mRNA from total RNA through A‐T base pairing with Oligo dT. Typically, rRNA removal is employed for transcriptome analysis.
- mRNA Fragmentation: The Illumina platform sequences short fragments, and the enriched mRNA consists of complete RNA sequences with an average length of several kilobases. Therefore, mRNA must be randomly fragmented. The addition of a fragmentation buffer allows for the random breakage of mRNA into ≈200 bp fragments.
- cDNA Synthesis Through Reverse Transcription: Under the action of reverse transcriptase and using random primers, the mRNA template was used to synthesize the first cDNA strand. During the second‐strand synthesis, dUTP was used in place of dTTP in the dNTPs reagent, resulting in the inclusion of A/U/C/G bases in the second cDNA strand.
- Adaptor Ligation: The double‐stranded cDNA structure has sticky ends. The End Repair Mix was added to blunt the ends, after which an A base was added to the 3′ end to facilitate the connection of the Y‐shaped adaptor.
- Digestion of the Second cDNA Strand with UNG Enzyme: Before PCR amplification, the second cDNA strand was digested using the UNG enzyme, ensuring that the library contained only the first cDNA strand.
- Illumina HiSeq Sequencing: 1) Library enrichment and PCR amplification for 15 cycles; 2) Quantification with TBS380 (Picogreen) and pooling samples according to data proportions for sequencing; 3) Bridge PCR amplification on the cBot to generate clusters; 4) Sequencing on the Illumina HiSeq platform, with 2×150 bp / 300 bp reads.

### H_2_S Detection

The classic lead acetate reactivity test was first used for detecting the presence of H_2_S. An appropriate amount of lead(II) acetate was dissolved in distilled water to create a lead acetate solution with a concentration of 2% for the test. A piece of filter paper was cut and soaked in the lead acetate solution until fully saturated. The paper was carefully removed from the solution and allowed to dry in a well‐ventilated area away from any H_2_S sources. Subsequently, the dried filter paper was placed over the surface of a 12‐well plate containing bacterial suspensions, and the bacteria were incubated at 37 °C for 24 h. Afterward, the color change of the filter paper was observed. The color intensity of the filter paper was finally compared to the standard. The WSP5 H_2_S‐nucleophilic substitution‐cyclization‐based fluorescent probe was then used to detect and quantify H_2_S in biological systems.

### Gene Knockout

The homologous recombination arms upstream and downstream of the CSE gene were amplified from the *Pseudomonas aeruginosa* (PAO1) genome using high‐fidelity DNA polymerase. The gentamicin resistance gene (Gm) was amplified from the pJQ200SK plasmid. The upstream and downstream homologous recombination arms of the CSE gene were connected to the Gm resistance gene using fusion PCR technology, yielding the complete target fragment ΔCSE::Gm (upstream homologous arm‐gentamicin resistance gene‐downstream homologous arm). This fragment was then cloned into the suicide plasmid pCVD442, generating the target plasmid pCVD442‐ΔCSE::Gm. Through electroporation, pCVD442‐ΔCSE::Gm was transformed into Escherichia coli β2155, resulting in the donor strain β2155/pCVD442‐ΔCSE::Gm. Conjugation experiments were performed between the donor strain β2155/pCVD442‐ΔCSE::Gm and the *Pseudomonas aeruginosa* recipient strain, and gentamicin‐resistant *Pseudomonas aeruginosa* clones were selected on gentamicin plates. These clones had the target plasmid integrated into their genome and were designated as PAO1/pCVD442‐ΔCSE::Gm. Several PAO1/pCVD442‐ΔCSE::Gm clones were streaked onto LB agar plates containing 10% sucrose and cultured until single colonies formed. PCR technology was utilized to screen for clones where the CSE gene was replaced by the Gm resistance gene, and these clones were named PAO1/ΔCSE::Gm. The helper plasmid pFLP3 was electroporated into the PAO1/ΔCSE::Gm strain, and the recombinase was heat‐induced to remove the Gm resistance gene from the genome, ultimately obtaining the markerless knockout strain PAO1/ΔCSE.

### Biological Electron Microscopy Observation

Bacterial cultures were grown to the desired growth phase. The bacterial cells were harvested by centrifugation, and the pellet was gently washed and resuspended in a suitable buffer. The bacterial suspension was fixed with 2.5% glutaraldehyde for 2 h. The bacteria were subjected to SEM (SIGMA 500, ZEISS, Oberkochen, Baden‐Württemberg, Germany) observation after being dehydrated in a gradient. For transmission electron microscopy (TEM) observation, the fixed bacterial cells were washed with a buffer and then treated with a secondary osmium tetroxide fixative for another 2 h. The fixed and post‐fixed bacterial cells were washed and then dehydrated through a graded series of ethanol, starting with a low concentration (30%) and incrementally increasing to a higher concentration (100%). The dehydrated bacterial cells were infiltrated with the epoxy by incubating the sample in a series of resin dilutions, gradually increasing the resin concentration. Once fully infiltrated, the sample was embedded in pure resin and allowed to polymerize at 60 °C for 48 h. The polymerized resin block containing the bacterial cells was trimmed and sectioned using an ultramicrotome. Ultrathin sections (50–90 nm thick) were collected on copper grids for examination. The ultrathin sections on grids were then treated with uranyl acetate. The bacterial members were finally observed by a biological TEM (HT7800, Hitachi, Tokyo, Japan).

### Preparation of Biofilm and Crystal Violet Staining

A 1 mL aliquot of the overnight culture, adjusted to 10^7^ CFU mL^−1^, was dispensed into each well of a 24‐well plate. Following incubation for 48 h at 37 °C (with daily medium replenishment), non‐adherent cells were removed by phosphate‐buffered saline (PBS) washing. The resultant biofilm was then harvested for biomass quantification via crystal violet staining, with absorbance measured at 590 nm using a microplate reader (SpectraMax i3, Molecular Devices, San Francisco, CA, USA).

### Growth Kinetic Curves

Bacterial growth kinetics were monitored using an automated growth analysis system (Tecan, Infinite Eplex, Mannedorf, Switzerland). Overnight cultures of strains, grown at 37 °C, were diluted 1:500 in fresh medium and aerated at 37 °C to mid‐exponential phase (10^8^ CFU mL^−1^). Cultures were then diluted 1:100 in LB broth. Triplicate 200 µL aliquots of each mixture were loaded into 96‐well plates. Growth at 37 °C was automatically tracked by recording OD_600_ at designated intervals. Data represent mean values of triplicate measurements.

### ROS Scavenging Assays

The cultured bacteria were centrifuged (6000 rpm, 5 min), the supernatant was discarded, and the pellet was washed twice with sterile physiological saline. The bacterial concentration was then adjusted to an appropriate density (OD_600_ = 0.5) for subsequent use. For the general ROS scavenging assay, the prepared bacterial suspension was mixed with DCFH‐DA solution (10 µmol L^−1^; Reactive Oxygen Species Assay Kit, cat# S0033, Beyotime, China) and incubated in the dark for 30 min. This allows DCFH‐DA to permeate the bacterial cells, where it is hydrolyzed by intracellular esterases to form DCFH. Subsequently, the mixture was co‐incubated with H_2_O_2_ solution (1 mm) for 10 min, enabling the reaction of DCFH with ROS to generate the fluorescent compound DCF. Fluorescence intensity was measured using a microplate reader (SpectraMax i3, Molecular Devices, San Francisco, CA, USA) at an excitation wavelength of 488 nm and an emission wavelength of 525 nm. The bacterial scavenging capacity for general ROS was calculated based on the change in fluorescence intensity. Fluorescence intensity was also visualized using fluorescence microscopy (IFM, IX73, Olympus, Tokyo, Japan). For singlet oxygen (^1^O_2_) scavenging assay, For singlet oxygen (^1^O_2_) scavenging assay, the 1,3‐diphenylisobenzofuran (DPBF) solution (100 µg mL^−1^) with bacterial suspension was mixed with the singlet oxygen‐generating photosensitizer Chlorin e6 (200 µg mL^−1^) and irradiated with a 660 nm laser for 10 min. A blank control group without bacteria was included. The absorbance of each group was measured at a specific wavelength (418 nm) using the microplate reader (SpectraMax i3). ^1^O_2_ causes a decrease in DPBF absorbance, and the bacterial scavenging capacity for ^1^O_2_ was calculated based on the change in absorbance. For hydroxyl radical (•OH) scavenging assay, a 5,5‐dimethyl‐1‐pyrroline‐N‐oxide (DMPO) solution (50 mm) was prepared as a spin‐trapping agent for •OH. The bacterial suspension was mixed with the DMPO solution and a •OH‐generating system (FeSO_4_‐H_2_O_2_). A positive control group without bacteria was also prepared. Samples were loaded into an electron spin resonance (ESR) spectrometer (Magnettech ESR5000, Bruker, Billerica, MA, USA), and the ESR spectra were recorded. The scavenging effect of bacteria on hydroxyl radicals was analyzed by comparing the signal intensities between the groups.

### In Vitro Antibacterial Tests

All the antibacterial experiments were conducted using the plate counting method. For the ^1^O_2_ antibacterial assay, a bacterial suspension (10^8^ CFU mL^−1^) was mixed with the ^1^O_2_‐generating photosensitizer Chlorin e6 (200 µg mL^−1^) and subsequently irradiated with a 660 nm laser for 10 min. For the •OH antibacterial assay, a bacterial suspension (10^8^ CFU mL^−1^) was mixed with a •OH‐generating system (FeSO_4_‐H_2_O_2_) for 10 min. Subsequently, the treated bacterial suspensions were diluted 1000‐fold with LB broth. A 20 µL aliquot of each dilution was then spread onto LB agar plates, followed by incubation at 37 °C for 24 h. The antibacterial rate was finally assessed by enumerating colony‐forming units (CFUs).

### Statistical Analysis

The data from all the experiments were analyzed by mean values ± standard deviations (SD) with n ≥ 3. The statistical analyses were performed by GraphPad Prism software, employing a one‐way analysis of variance (ANOVA) program followed by Tukey's multiple comparisons test for multiple comparisons and a two‐sample Student's *t*‐test for two groups. Additionally, P values of *p <0.05, **p <0.01, ***p <0.001, and ****p <0.0001 were considered as statistically significant.

## Conflict of Interest

The authors declare no conflict of interest.

## Supporting information



Supporting Information

## Data Availability

The data that support the findings of this study are available from the corresponding author upon reasonable request.
