# Supplementary material for: Biosynthesis of H2S and Siderophores Targeting Gram‐Negative Bacterial Resistance to Reactive Oxygen Species
Source: Adv Sci (Weinh). 2025 Sep 15;12(45):e05748. doi: 10.1002/advs.202505748 (PMC12677624; doi:10.1002/advs.202505748)
Supplement: Supplementary file 1 — Supporting Information [file ADVS-12-e05748-s001.docx]

Supporting Information

Biosynthesis of H_2_S and siderophores targeting Gram-negative bacterial resistance to reactive oxygen species

Congyang Mao†, Wanyu Jin†, Yiming Xiang, Yizhou Zhu, Jun Wu, Xiangmei Liu*, Shuilin Wu*, Wei Qiao, Kenneth M. C. Cheung, Kelvin Wai Kwok Yeung*

**Table of contents**

**Table S1.** The exact H_2_O_2_ concentrations (μg mL^-1^) for all reported MIC values for Gram-negative bacteria, including *P. aeruginosa*, *E. coli*, and *Klebsiella pneumoniae*.

**Table S2.** The exact H_2_O_2_ concentrations (μg mL^-1^) for all reported MIC values for *S. aureus*, USA300.

**Figure S1.** Protocol of bacterial development resistance to ROS. Ten consecutive passages of bacterial cultivation were performed in the presence of varying H_2_O_2_ concentrations. Within the 96-well plates, the H_2_O_2_ concentration underwent a sequential reduction by 50% from column 1 to 12, where the concentration of 1F-H was equivalent to half of 12A-C and subsequently decreased by half in a sequential manner; this process was repeated three times for each concentration.

**Figure S2.** Evolution of ROS resistance in *E. coli* K-12. Photographs of the 96-well plates containing different passages (PN indicates passage N) of bacteria after cocultivation with H_2_O_2_ (Concentration decreasing 23 times in half from left to right, top to bottom) at 37 ^o^C for 24 h. The H_2_O_2_ concentration within the clear wells directly neighboring the turbid wells was recorded as the MIC. The treated bacteria that survived in these turbid wells with the highest H_2_O_2_ concentration were collected as the succeeding bacterial passage, and then subjected to a rechallenge employing an analogous methodology until P10 was reached.

**Figure S3.** Evolution of ROS resistance in *Klebsiella pneumoniae*. Photographs of the 96-well plates containing different passages of bacteria after cocultivation with H_2_O_2_ (Concentration decreasing 23 times in half from left to right, top to bottom) at 37 ^o^C for 24 h. The H_2_O_2_ concentration within the clear wells directly neighboring the turbid wells was recorded as the MIC. The treated bacteria that survived in these turbid wells with the highest H_2_O_2_ concentration were collected as the succeeding bacterial passage, and then subjected to a rechallenge employing an analogous methodology until P10 was reached.

**Figure S4.** Enhanced *cse* and *cbs* genes-mediated KEGG pathways. Chord plot illustrating the most significant enrichment in the upregulated KEGG pathways in P10 *P. aeruginosa* bacterial strain compared to the WT (P1) bacterial strain. (Fisher exact test was employed, adjusted *P* < 0.05)

**Figure S5.** Protocol of H_2_S by the classic lead acetate reactivity test. A filter paper saturated in a 2% lead acetate solution is situated above a well plate containing a bacterial suspension. The H_2_S generated by the bacteria subsequently undergoes a reaction with the lead acetate, resulting in the formation of a brown precipitate comprised of lead sulfide.

**Figure S6.** Molecular structural formula of PVD.

**Figure S7.** Enhanced biosynthesis of PCH and its regulated Fe^3+^ uptake. a,b) Relative expression of genes related to biosynthesis of a) PCH and b) uptake of PCH-Fe^3+^ complex in the WT (P1) and P10 bacterial strains. Data were obtained from independent samples (n = 3). Error bars represent the mean ± standard deviation, with significance levels indicated as **p* < 0.05, ***p* < 0.01, and ****p* < 0.001. A two-sample Student *t*-test was utilized for the statistical analysis.

**Figure S8.** SEM images of the WT (P1) and P10 bacteria.

**Figure S9.** ROS defense barriers mediated by enhanced tyrosine metabolism and biofilm formation. a) Relative expression of *phhc* gene related to tyrosine metabolism in the WT (P1) and P10 bacterial strains. Data were obtained from independent samples (n = 3). Error bars represent the mean ± standard deviation, with significance levels indicated as **p* < 0.05, ***p* < 0.01, and ****p* < 0.001. A two-sample Student *t*-test was utilized for the statistical analysis. b) A crucial metabolic pathway pertaining to tyrosine encompasses its transformation into homogentisic acid, facilitated by the catalytic action of tyrosine aminotransferase. c,d) Relative expression of genes related to c) biofilm biosynthesis and d) biofilm formation in the WT (P1) and P10 bacterial strains. Data were obtained from independent samples (n = 3). Error bars represent the mean ± standard deviation, with significance levels indicated as **p* < 0.05, ***p* < 0.01, and ****p* < 0.001. A two-sample Student *t*-test was utilized for the statistical analysis.

**Figure S10.** The evaluation of biofilm formation. a) Crystal violet staining of WT (P1) and P10 bacterial biofilms. b) Absorbance at 590 nm corresponding to crystal violet staining. Data were obtained from independent samples (n = 3). Error bars represent the mean ± standard deviation, with significance levels indicated as **p* < 0.05, ***p* < 0.01, and ****p* < 0.001. A two-sample Student *t*-test was utilized for the statistical analysis.

**Figure S11.** Growth kinetics of WT (P1) and P10 bacterial strains. Data were obtained from independent samples (n = 3).

**Figure S12.** Relative expression of *relA* gene related to bacterial stringent response in the WT (P1) and P10 bacterial strains. Data were obtained from independent samples (n = 3). Error bars represent the mean ± standard deviation, with significance levels indicated as **p* < 0.05, ***p* < 0.01, and ****p* < 0.001. A two-sample Student *t*-test was utilized for the statistical analysis.

**Figure S13.** Relative expression of genes related to pyocyanin virulence factor in the WT (P1) and P10 bacterial strains. Data were obtained from independent samples (n = 3). Error bars represent the mean ± standard deviation, with significance levels indicated as **p* < 0.05, ***p* < 0.01, and ****p* < 0.001. A two-sample Student *t*-test was utilized for the statistical analysis.

**Figure S14.** The evaluation of the scavenging ability of general ROS. a,b) a) The fluorescence images of oxidative stress after bacterial treatment with ROS and b) the corresponding fluorescence intensities. Data were obtained from independent samples (n = 3). Error bars represent the mean ± standard deviation, with significance levels indicated as **p* < 0.05, ***p* < 0.01, and ****p* < 0.001. A two-sample Student *t*-test was utilized for the statistical analysis.

**Figure S15.** The evaluation of the scavenging ability of ^1^O_2_. a,b) a) The absorbance of DPBF after different treatments and b) the corresponding ^1^O_2_ scavenging rate. Data were obtained from independent samples (n = 3). Error bars represent the mean ± standard deviation, with significance levels indicated as **p* < 0.05, ***p* < 0.01, and ****p* < 0.001. A two-sample Student *t*-test was utilized for the statistical analysis.

**Figure S16.** ESR spectra of •OH after different treatments.

**Figure S17. WT (P1) and P10 bacterial strains in response to ^1^O_2_ and •OH.** a,b) a)Visible colony units of P1 (left) and P1 treated by ^1^O_2_ and •OH for 10 min (right) and b) corresponding antibacterial ratio. c,d) c) Visible colony units of P10 (left) and P10 treated by ^1^O_2_ and •OH for 10 min (right) and d) corresponding antibacterial ratio. Data were obtained from independent samples (n = 3). Error bars represent the mean ± standard deviation, with significance levels indicated as **p* < 0.05, ***p* < 0.01, and ****p* < 0.001. A one-way analysis of variance with the Tukey multiple-comparisons test was utilized for the statistical analysis.

**Figure S18.** Evolution of ROS resistance in *S. aureus*, USA300. Photographs of the 96-well plates containing different passages (PN indicates passage N) of bacteria after cocultivation with H_2_O_2_ (Concentration decreasing 23 times in half from left to right, top to bottom) at 37 ^o^C for 24 h. The H_2_O_2_ concentration within the clear wells directly neighboring the turbid wells was recorded as the MIC. The treated bacteria that survived in these turbid wells with the highest H_2_O_2_ concentration were collected as the succeeding bacterial passage, and then subjected to a rechallenge employing an analogous methodology until P10 was reached.

**Figure S19.** The development of ROS resistance in Gram-positive bacteria *S. aureus* USA300 is depicted in response to H_2_O_2_. This is represented by a fold increase in MIC versus the passage number. All experiments were performed in triplicate, with a sample size of three (n=3).

**Table S1.** The exact H_2_O_2_ concentrations (μg mL^-1^) for all reported MIC values for Gram-negative bacteria, including *P. aeruginosa*, *E. coli*, and *Klebsiella pneumoniae*.

| Passages | P1 | P2 | P3 | P4 | P5 | P6 | P7 | P8 | P9 | P10 |
| --- | --- | --- | --- | --- | --- | --- | --- | --- | --- | --- |
| PAO1  MIC (μg mL^-1^) | 18.3 | 36.6 | 36.6 | 73.2 | 73.2 | 146.4 | 1172 | 2344 | 4688 | 4688 |
| *E. coli* K-12  MIC (μg mL^-1^) | 18.3 | 36.6 | 18.3 | 36.6 | 146.4 | 146.4 | 292.8 | 292.8 | 585.6 | 585.6 |
| *Klebsiella pneumoniae*  MIC (μg mL^-1^) | 18.3 | 36.6 | 36.6 | 146.4 | 146.4 | 146.4 | 292.8 | 292.8 | 292.8 | 585.6 |

**Table S2.** The exact H_2_O_2_ concentrations (μg mL^-1^) for all reported MIC values for *S. aureus*, USA300.

| Passages | P1 | P2 | P3 | P4 | P5 | P6 | P7 | P8 | P9 | P10 |
| --- | --- | --- | --- | --- | --- | --- | --- | --- | --- | --- |
| *S. aureus*, USA300  MIC (μg mL^-1^) | 9.15 | 9.15 | 18.3 | 9.15 | 9.15 | 18.3 | 18.3 | 18.3 | 36.6 | 36.6 |


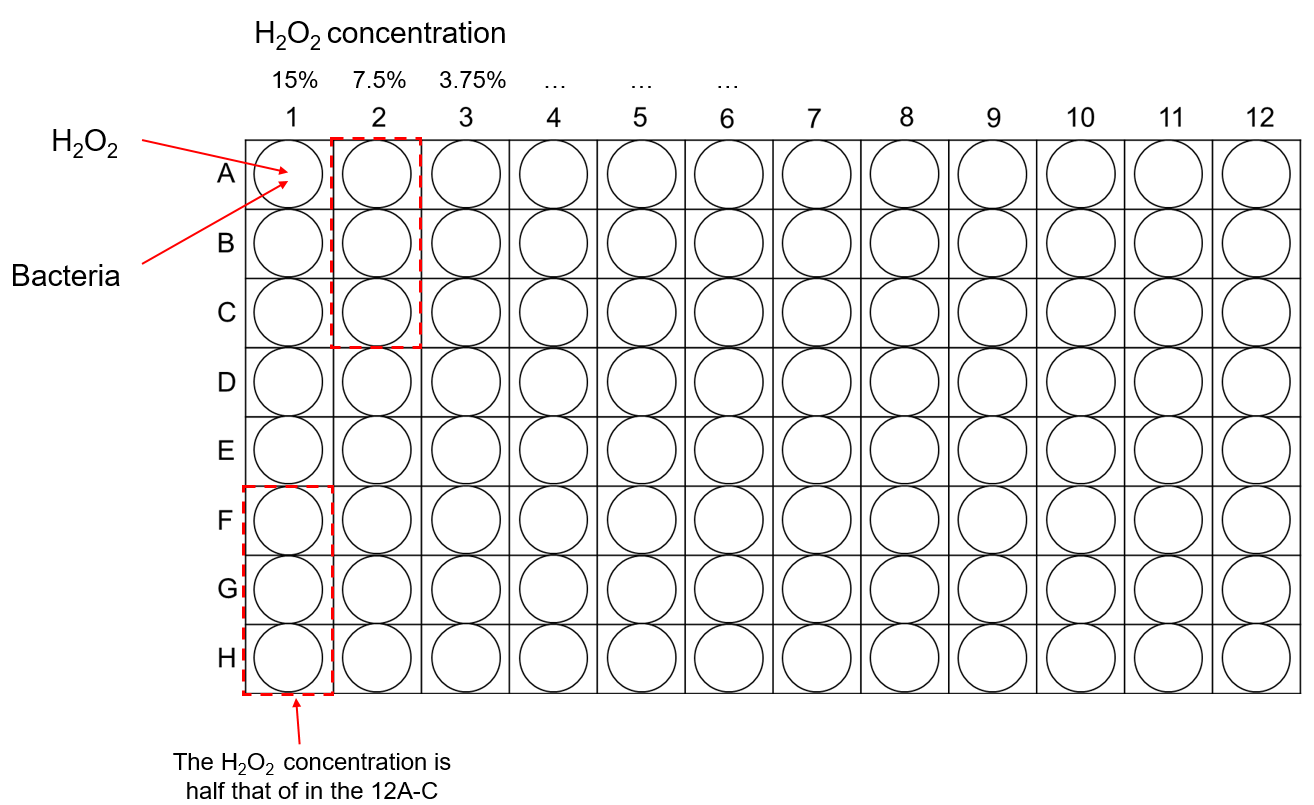


**Figure S1.** Protocol of bacterial development resistance to ROS. Ten consecutive passages of bacterial cultivation were performed in the presence of varying H_2_O_2_ concentrations. Within the 96-well plates, the H_2_O_2_ concentration underwent a sequential reduction by 50% from column 1 to 12, where the concentration of 1F-H was equivalent to half of 12A-C and subsequently decreased by half in a sequential manner; this process was repeated three times for each concentration.

**
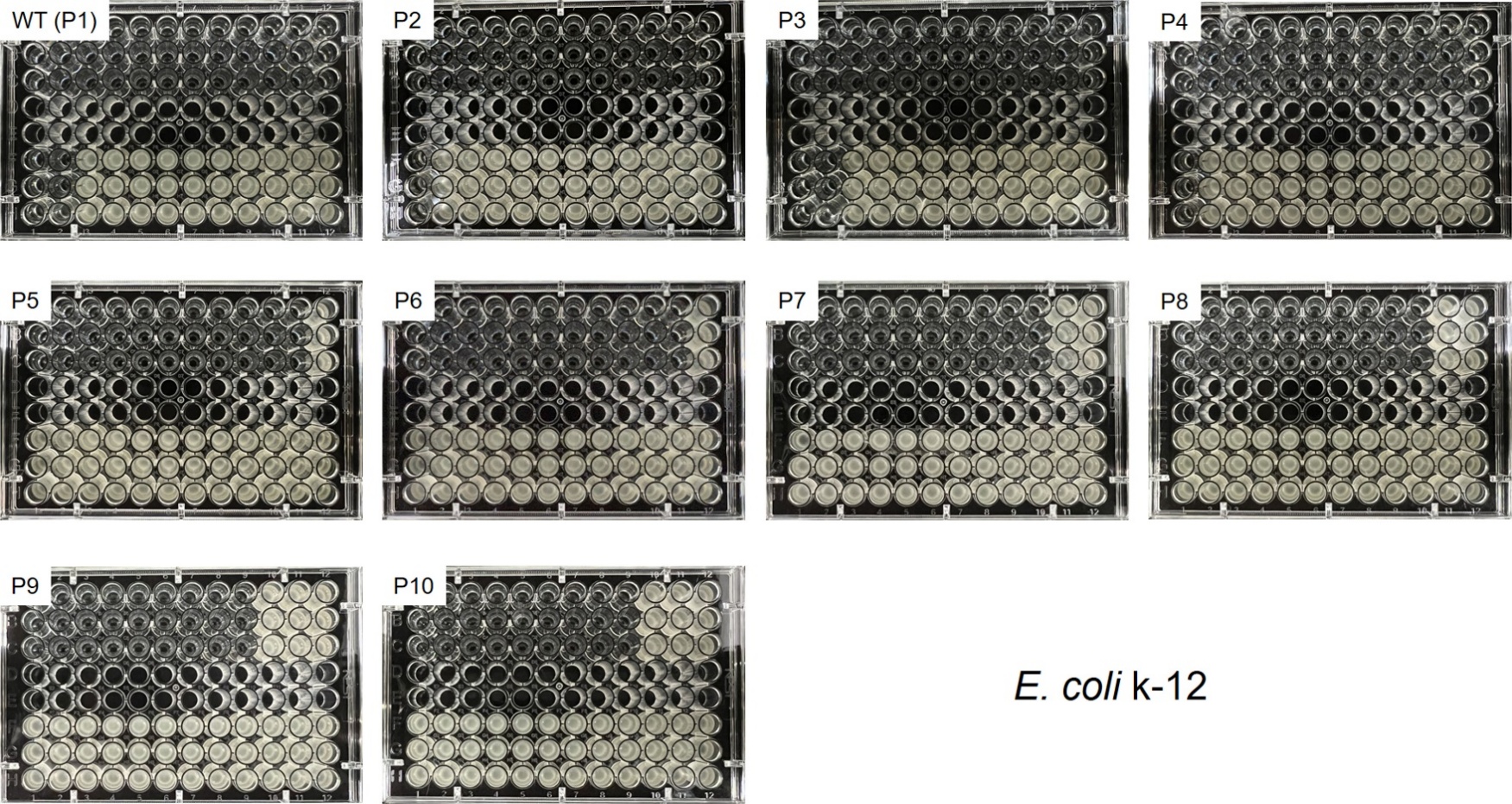
**

**Figure S2.** Evolution of ROS resistance in *E. coli* K-12. Photographs of the 96-well plates containing different passages (PN indicates passage N) of bacteria after cocultivation with H_2_O_2_ (Concentration decreasing 23 times in half from left to right, top to bottom) at 37 ^o^C for 24 h. The H_2_O_2_ concentration within the clear wells directly neighboring the turbid wells was recorded as the MIC. The treated bacteria that survived in these turbid wells with the highest H_2_O_2_ concentration were collected as the succeeding bacterial passage, and then subjected to a rechallenge employing an analogous methodology until P10 was reached.

**
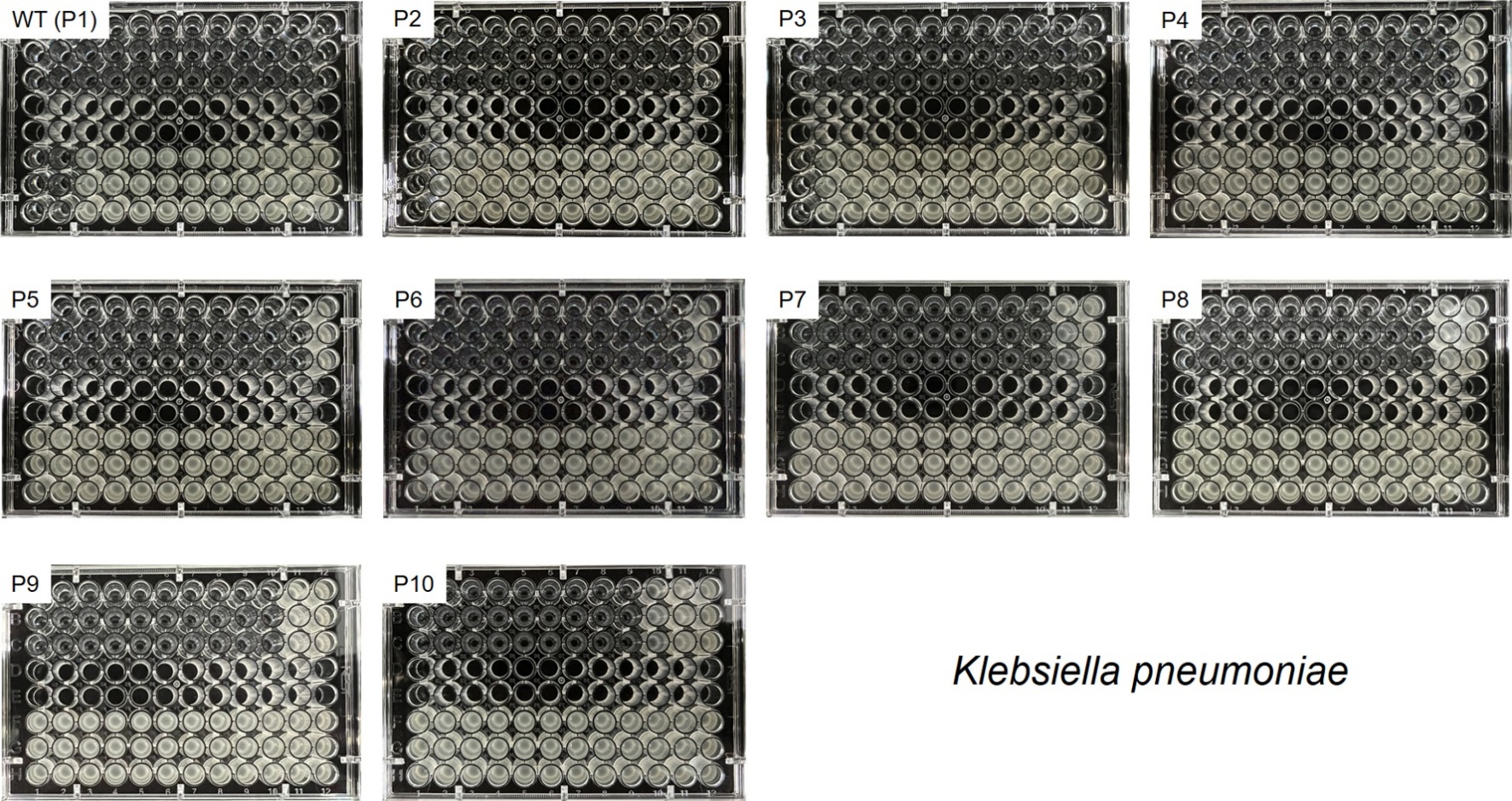
**

**Figure S3.** Evolution of ROS resistance in *Klebsiella pneumoniae*. Photographs of the 96-well plates containing different passages of bacteria after cocultivation with H_2_O_2_ (Concentration decreasing 23 times in half from left to right, top to bottom) at 37 ^o^C for 24 h. The H_2_O_2_ concentration within the clear wells directly neighboring the turbid wells was recorded as the MIC. The treated bacteria that survived in these turbid wells with the highest H_2_O_2_ concentration were collected as the succeeding bacterial passage, and then subjected to a rechallenge employing an analogous methodology until P10 was reached.

**
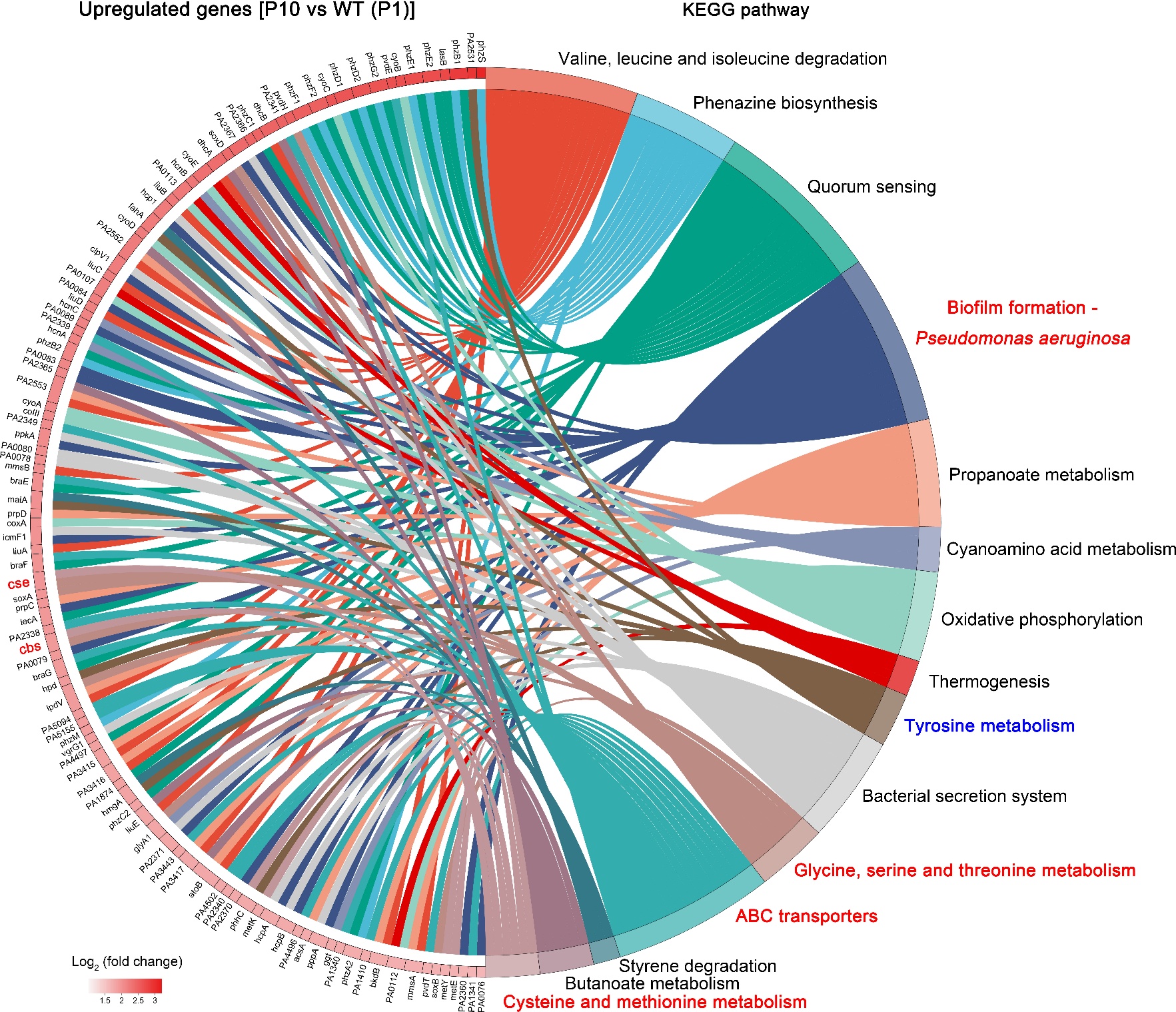
**

**Figure S4.** Enhanced *cse* and *cbs* genes-mediated KEGG pathways. Chord plot illustrating the most significant enrichment in the upregulated KEGG pathways in P10 *P. aeruginosa* bacterial strain compared to the WT (P1) bacterial strain. (Fisher exact test was employed, adjusted *P* < 0.05)

**
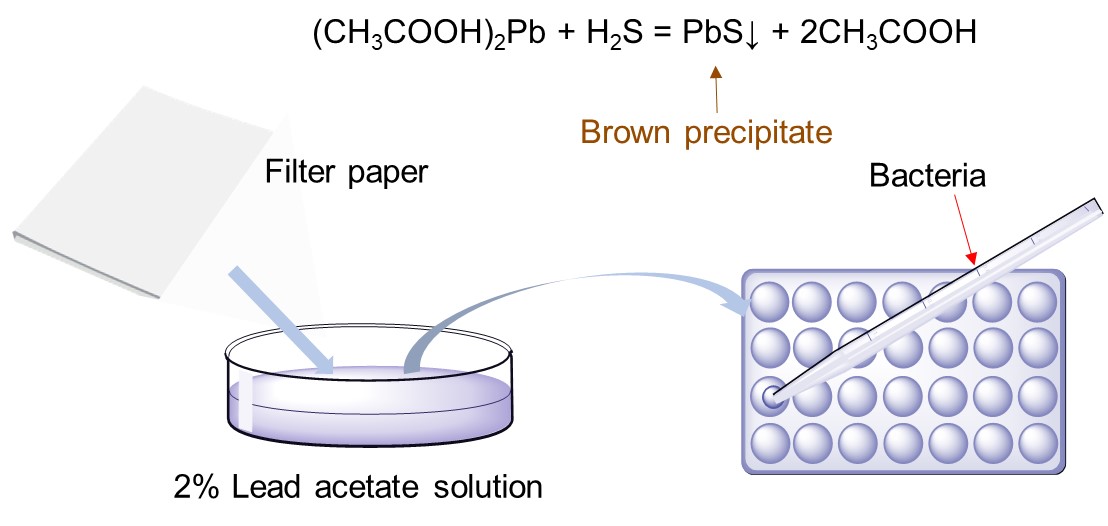
**

**Figure S5.** Protocol of H_2_S by the classic lead acetate reactivity test. A filter paper saturated in a 2% lead acetate solution is situated above a well plate containing a bacterial suspension. The H_2_S generated by the bacteria subsequently undergoes a reaction with the lead acetate, resulting in the formation of a brown precipitate comprised of lead sulfide.

**
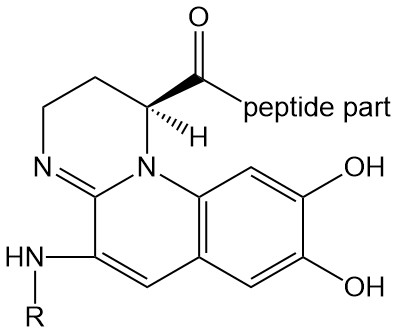
**

**Figure S6.** Molecular structural formula of PVD.

**
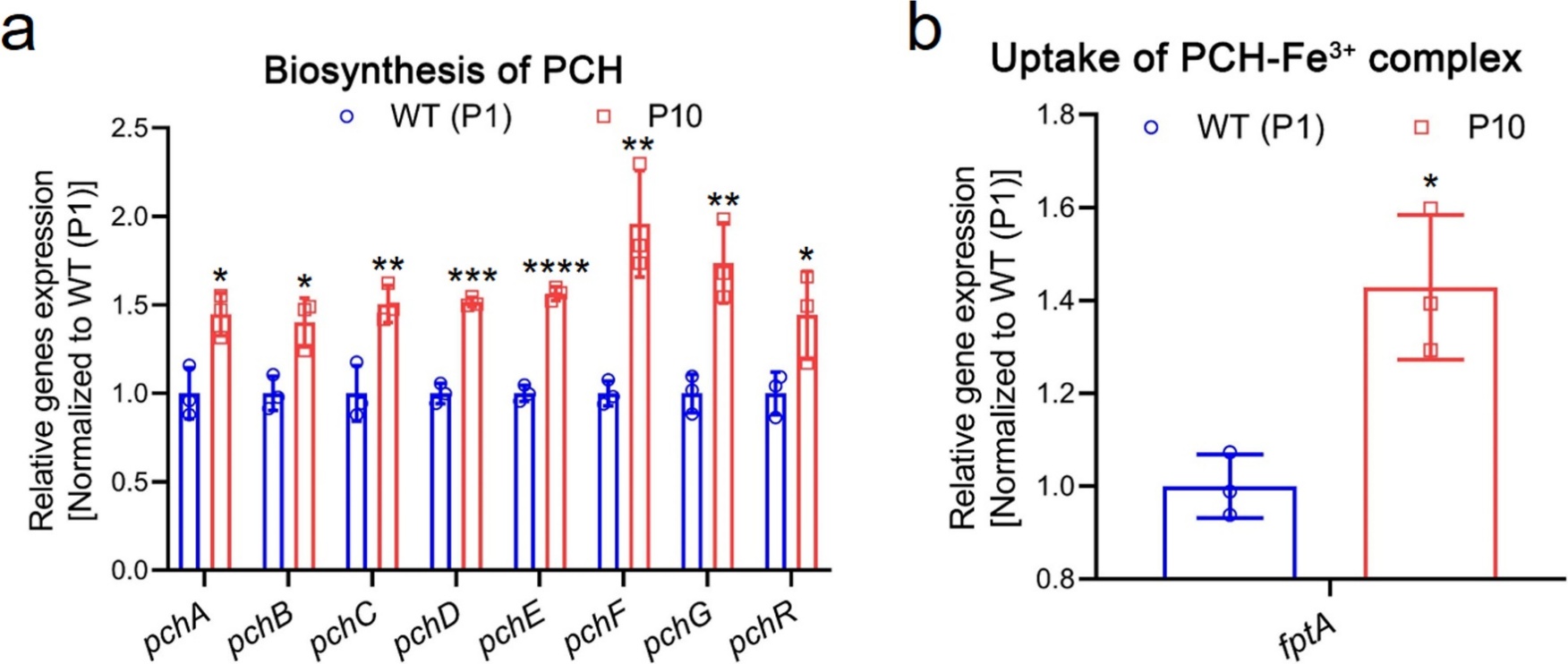
**

**Figure S7.** Enhanced biosynthesis of PCH and its regulated Fe^3+^ uptake. a,b) Relative expression of genes related to biosynthesis of a) PCH and b) uptake of PCH-Fe^3+^ complex in the WT (P1) and P10 bacterial strains. Data were obtained from independent samples (n = 3). Error bars represent the mean ± standard deviation, with significance levels indicated as **p* < 0.05, ***p* < 0.01, and ****p* < 0.001. A two-sample Student *t*-test was utilized for the statistical analysis.

**
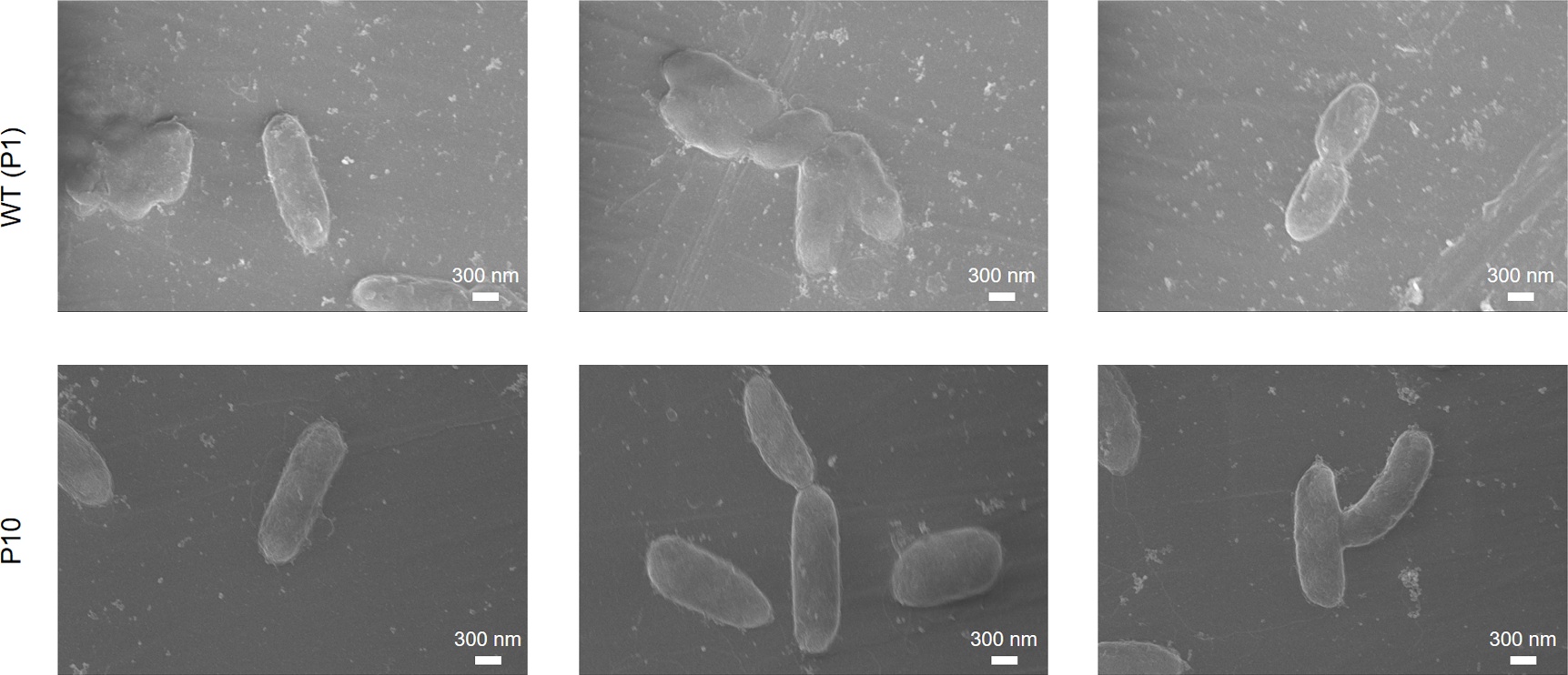
**

**Figure S8.** SEM images of the WT (P1) and P10 bacteria.


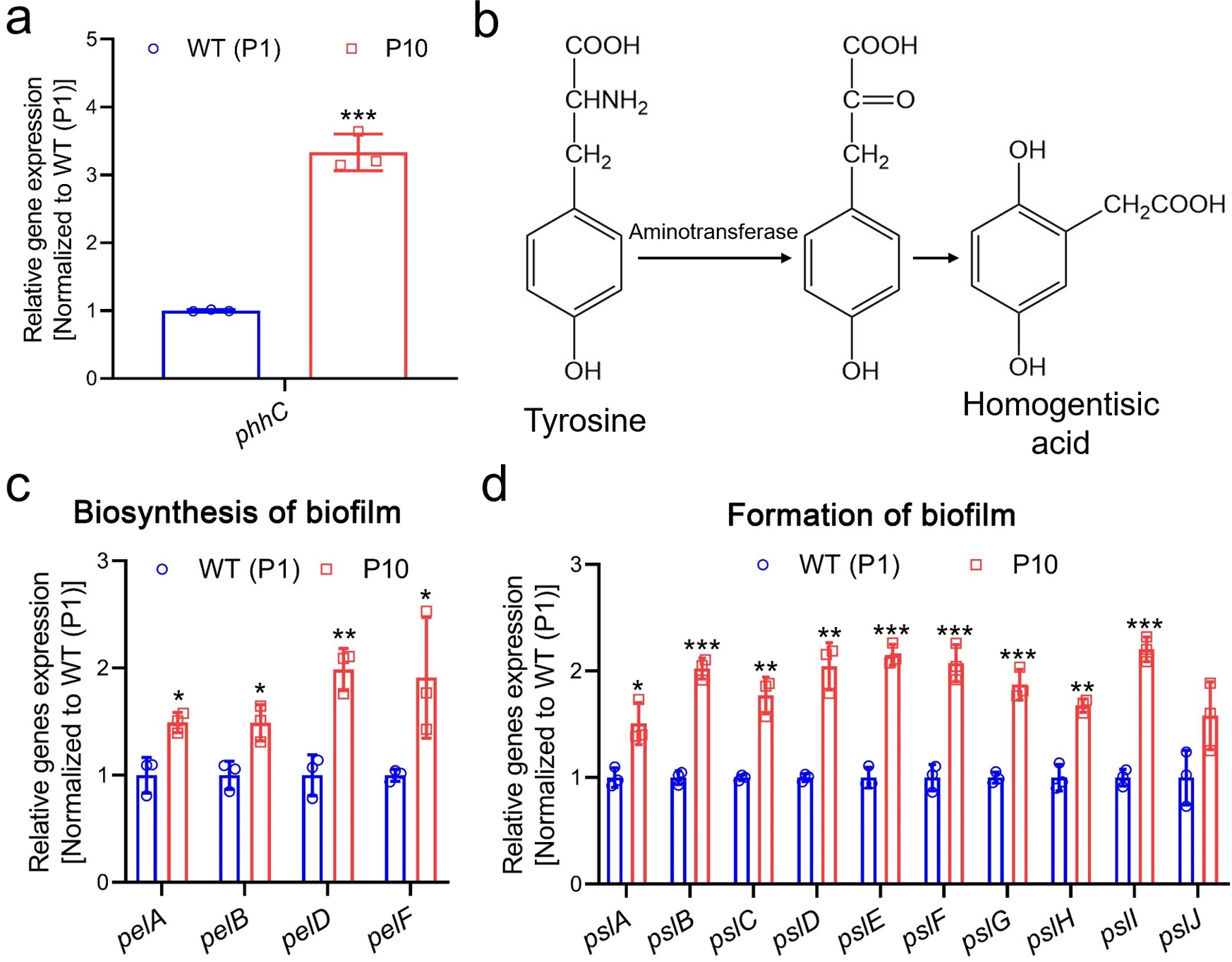


**Figure S9.** ROS defense barriers mediated by enhanced tyrosine metabolism and biofilm formation. a) Relative expression of *phhc* gene related to tyrosine metabolism in the WT (P1) and P10 bacterial strains. Data were obtained from independent samples (n = 3). Error bars represent the mean ± standard deviation, with significance levels indicated as **p* < 0.05, ***p* < 0.01, and ****p* < 0.001. A two-sample Student *t*-test was utilized for the statistical analysis. b) A crucial metabolic pathway pertaining to tyrosine encompasses its transformation into homogentisic acid, facilitated by the catalytic action of tyrosine aminotransferase. c,d) Relative expression of genes related to c) biofilm biosynthesis and d) biofilm formation in the WT (P1) and P10 bacterial strains. Data were obtained from independent samples (n = 3). Error bars represent the mean ± standard deviation, with significance levels indicated as **p* < 0.05, ***p* < 0.01, and ****p* < 0.001. A two-sample Student *t*-test was utilized for the statistical analysis.

**
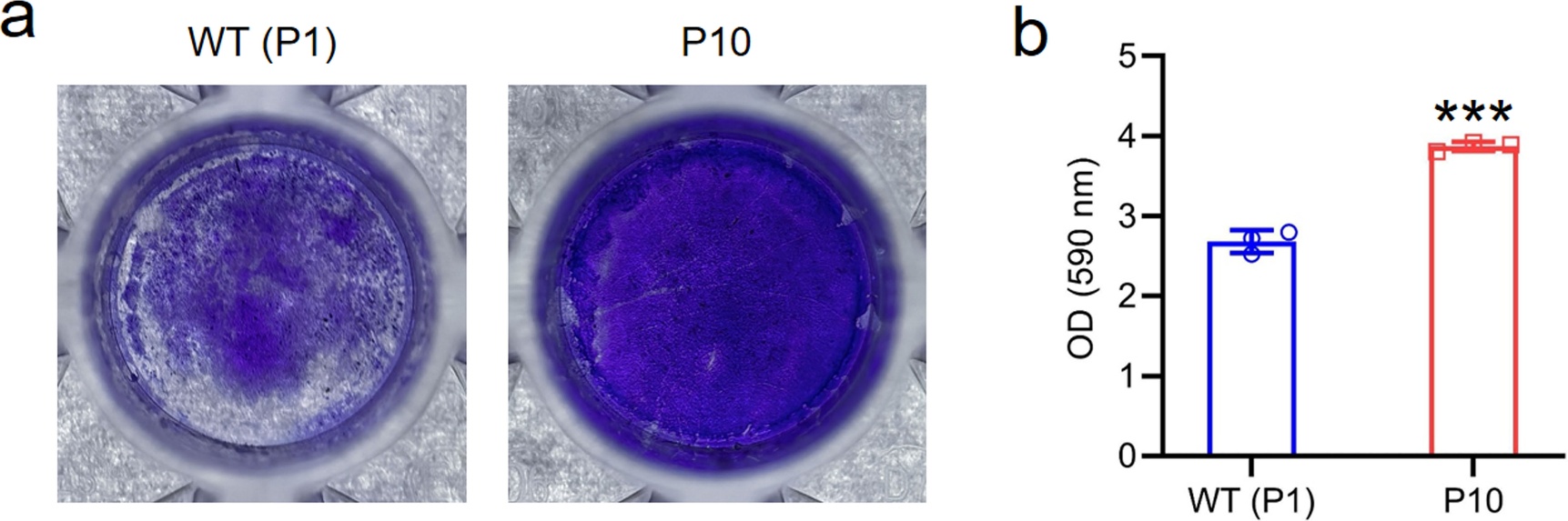
**

**Figure S10.** The evaluation of biofilm formation. a) Crystal violet staining of WT (P1) and P10 bacterial biofilms. b) Absorbance at 590 nm corresponding to crystal violet staining. Data were obtained from independent samples (n = 3). Error bars represent the mean ± standard deviation, with significance levels indicated as **p* < 0.05, ***p* < 0.01, and ****p* < 0.001. A two-sample Student *t*-test was utilized for the statistical analysis.

**
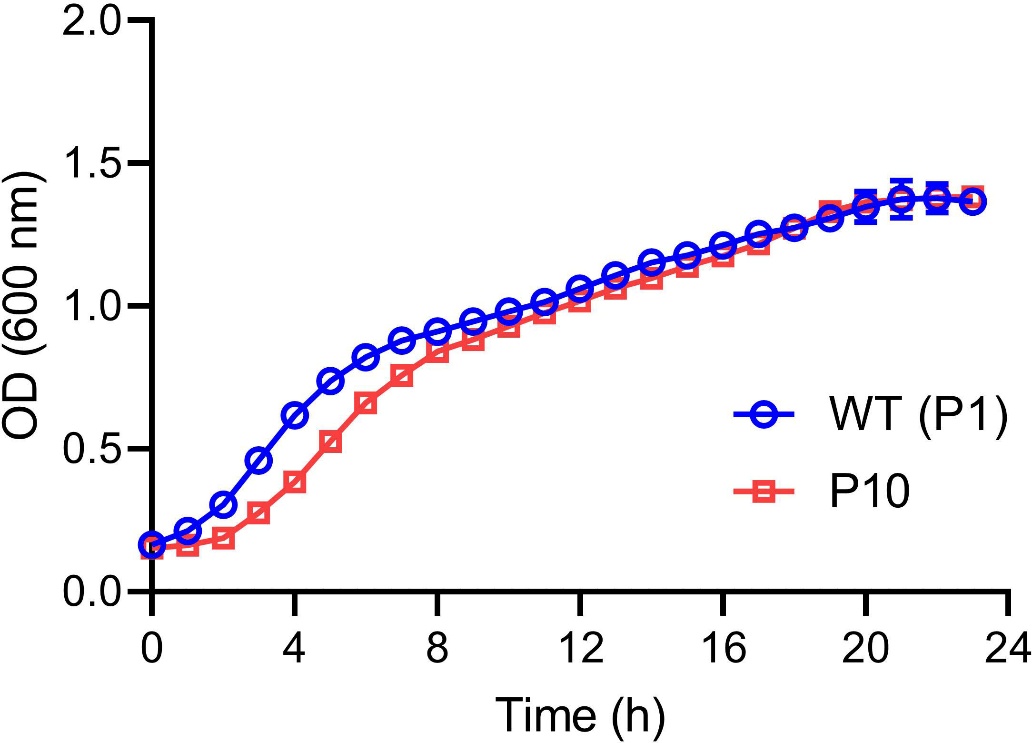
**

**Figure S11.** Growth kinetics of WT (P1) and P10 bacterial strains. Data were obtained from independent samples (n = 3).

**
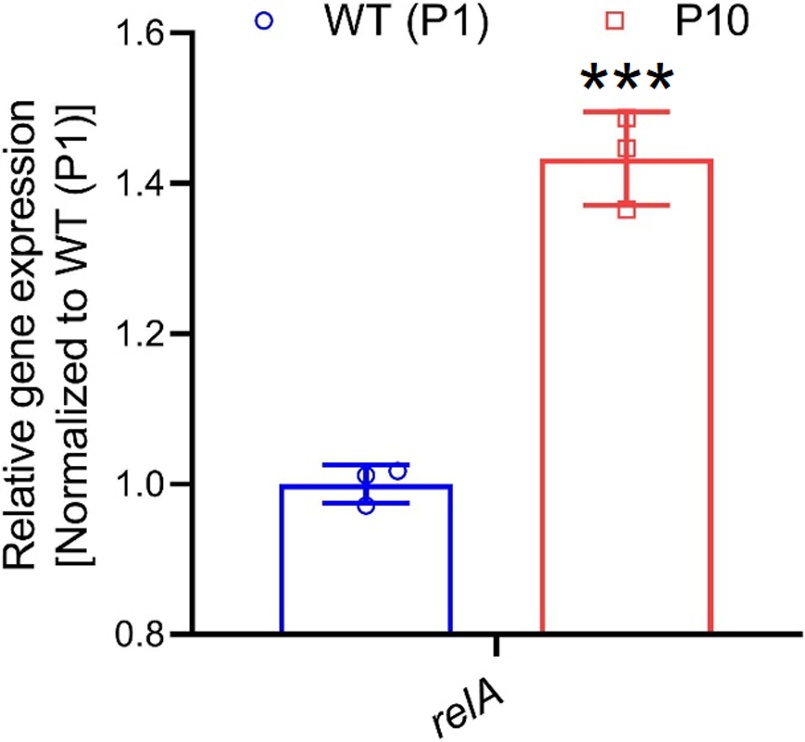
**

**Figure S12.** Relative expression of *relA* gene related to bacterial stringent response in the WT (P1) and P10 bacterial strains. Data were obtained from independent samples (n = 3). Error bars represent the mean ± standard deviation, with significance levels indicated as **p* < 0.05, ***p* < 0.01, and ****p* < 0.001. A two-sample Student *t*-test was utilized for the statistical analysis.

**
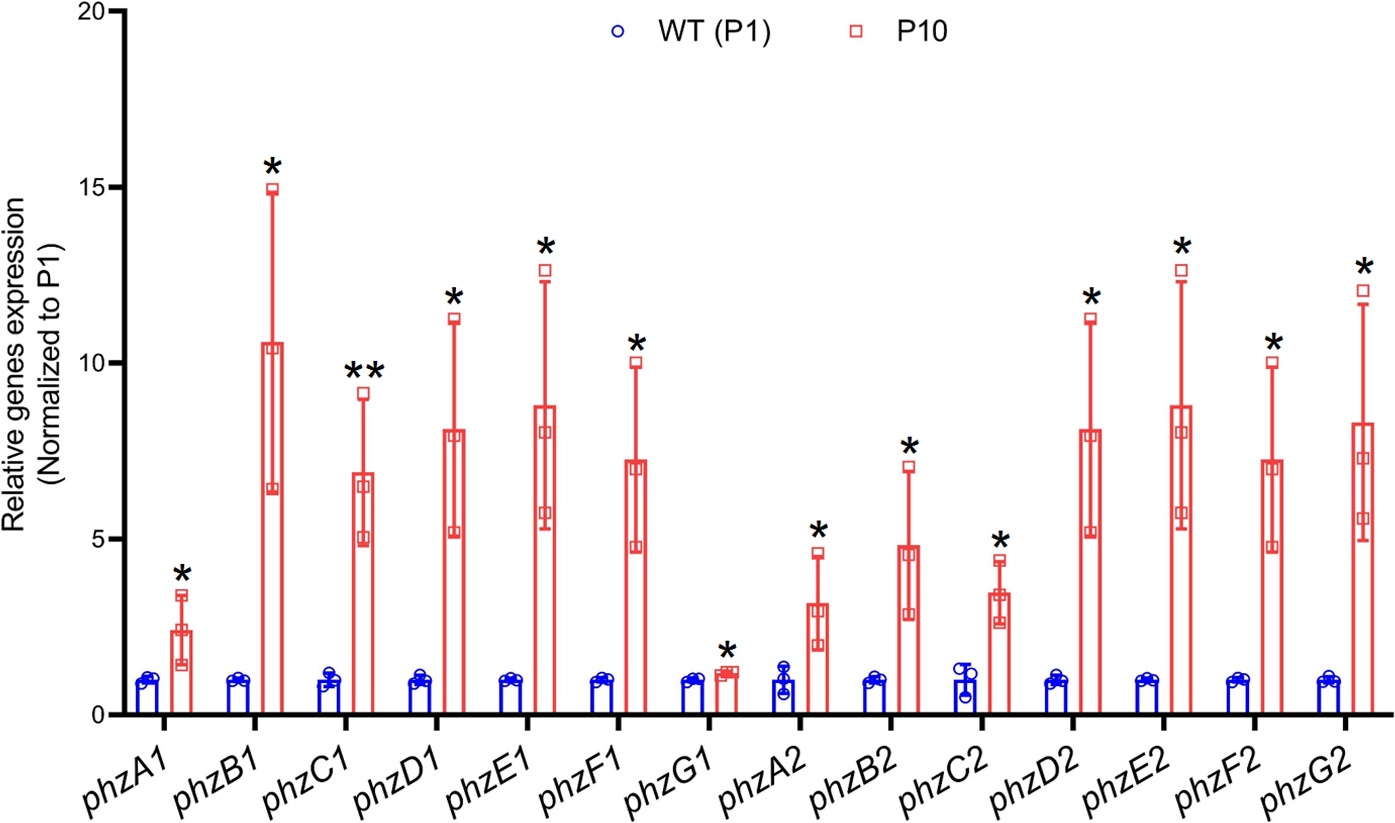
**

**Figure S13.** Relative expression of genes related to pyocyanin virulence factor in the WT (P1) and P10 bacterial strains. Data were obtained from independent samples (n = 3). Error bars represent the mean ± standard deviation, with significance levels indicated as **p* < 0.05, ***p* < 0.01, and ****p* < 0.001. A two-sample Student *t*-test was utilized for the statistical analysis.

**
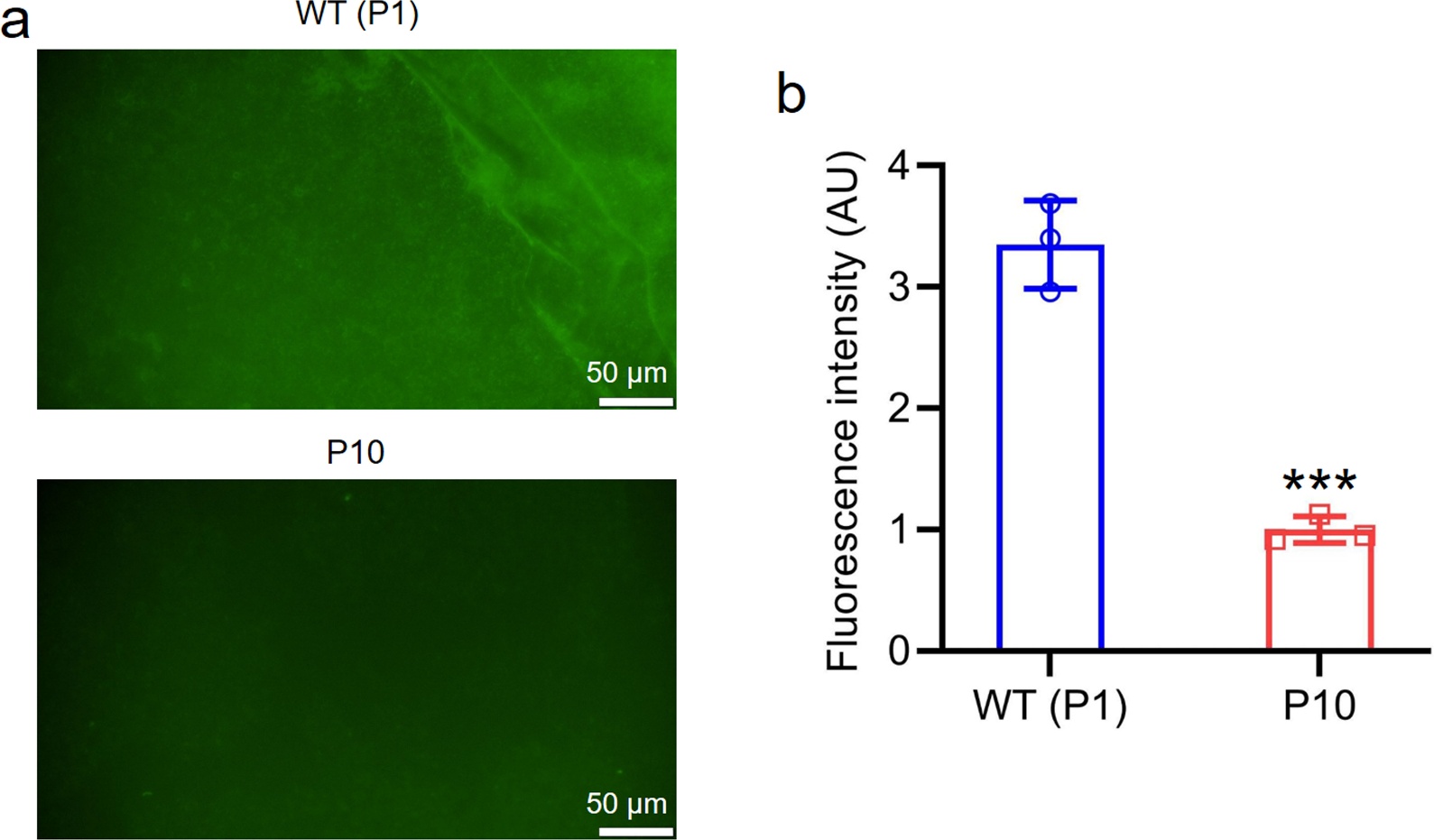
**

**Figure S14.** The evaluation of the scavenging ability of general ROS. a,b) a) The fluorescence images of oxidative stress after bacterial treatment with ROS and b) the corresponding fluorescence intensities. Data were obtained from independent samples (n = 3). Error bars represent the mean ± standard deviation, with significance levels indicated as **p* < 0.05, ***p* < 0.01, and ****p* < 0.001. A two-sample Student *t*-test was utilized for the statistical analysis.

**
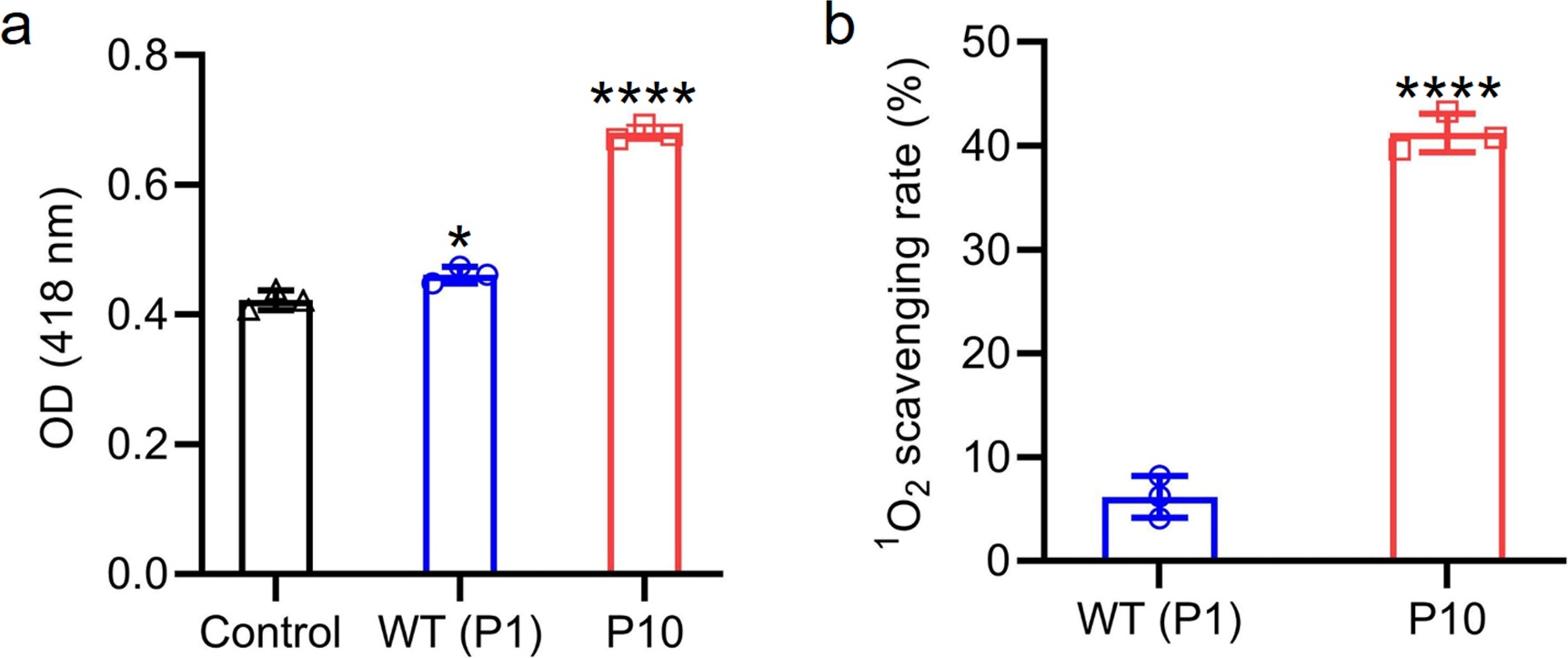
**

**Figure S15.** The evaluation of the scavenging ability of ^1^O_2_. a,b) a) The absorbance of DPBF after different treatments and b) the corresponding ^1^O_2_ scavenging rate. Data were obtained from independent samples (n = 3). Error bars represent the mean ± standard deviation, with significance levels indicated as **p* < 0.05, ***p* < 0.01, and ****p* < 0.001. A two-sample Student *t*-test was utilized for the statistical analysis.

**
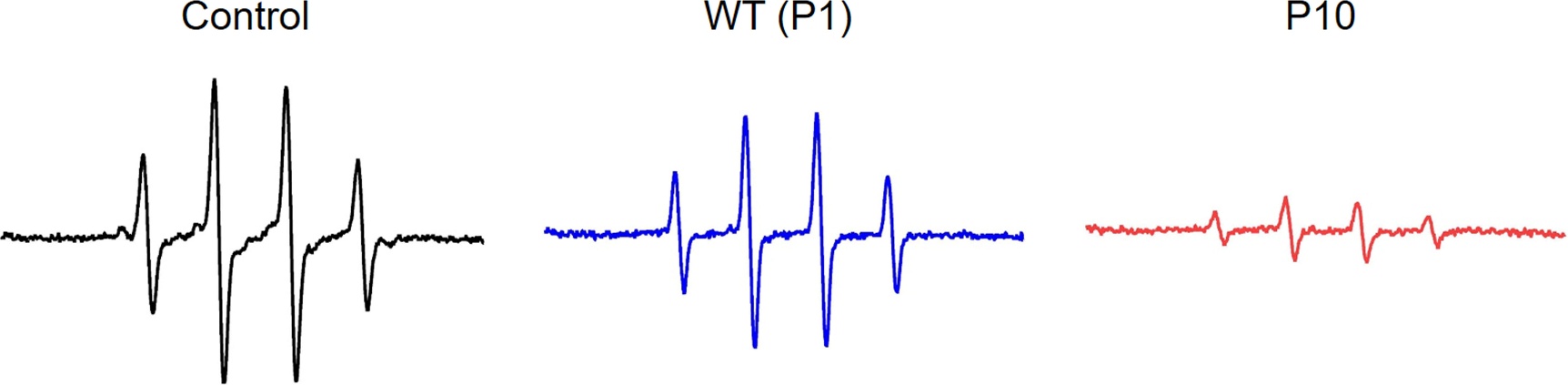
**

**Figure S16.** ESR spectra of •OH after different treatments.


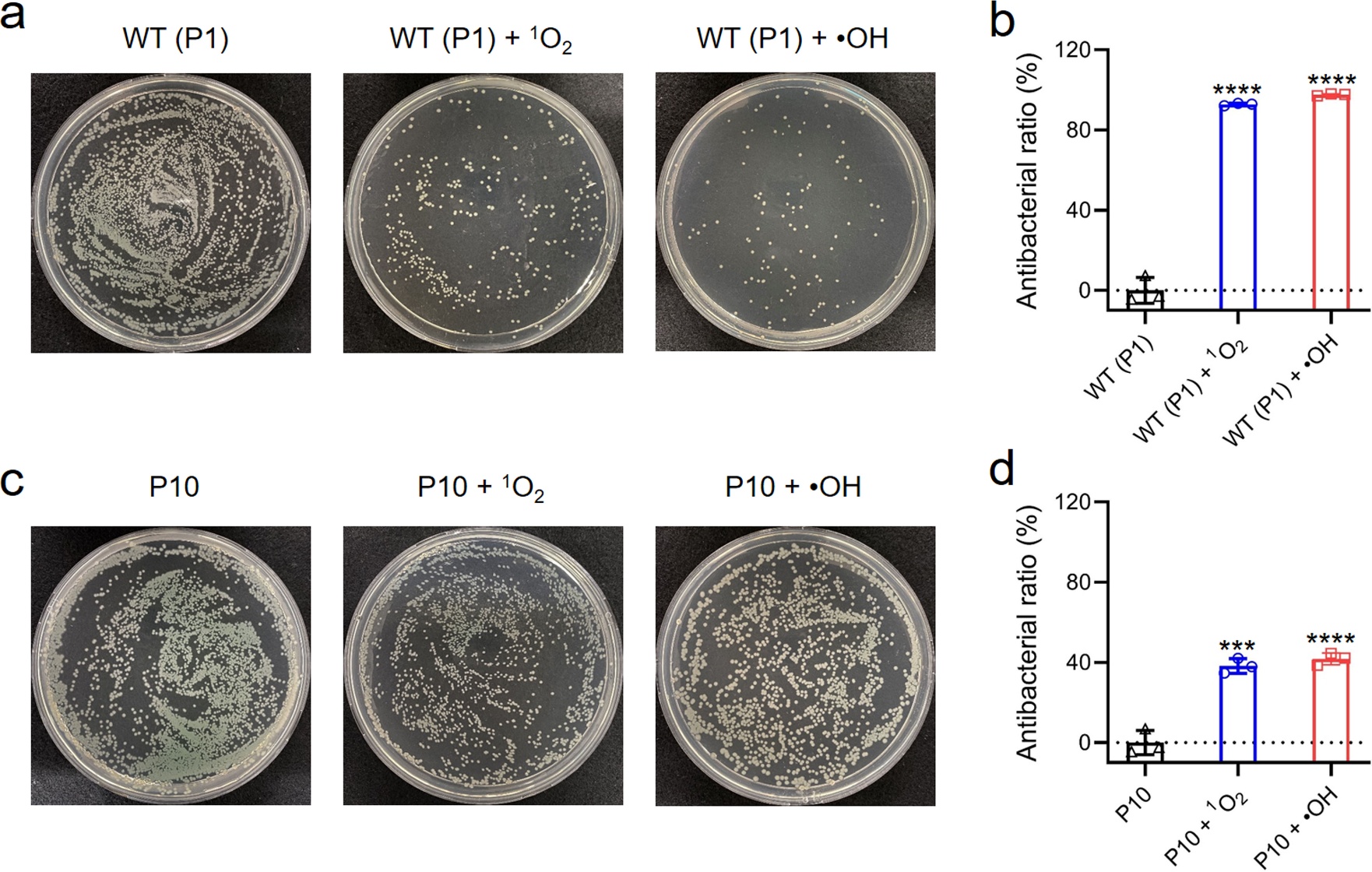


**Figure S17.** **WT (P1) and P10 bacterial strains in response to ^1^O_2_ and •OH.** a,b) a)Visible colony units of P1 (left) and P1 treated by ^1^O_2_ and •OH for 10 min (right) and b) corresponding antibacterial ratio. c,d) c) Visible colony units of P10 (left) and P10 treated by ^1^O_2_ and •OH for 10 min (right) and d) corresponding antibacterial ratio. Data were obtained from independent samples (n = 3). Error bars represent the mean ± standard deviation, with significance levels indicated as **p* < 0.05, ***p* < 0.01, and ****p* < 0.001. A one-way analysis of variance with the Tukey multiple-comparisons test was utilized for the statistical analysis.

**
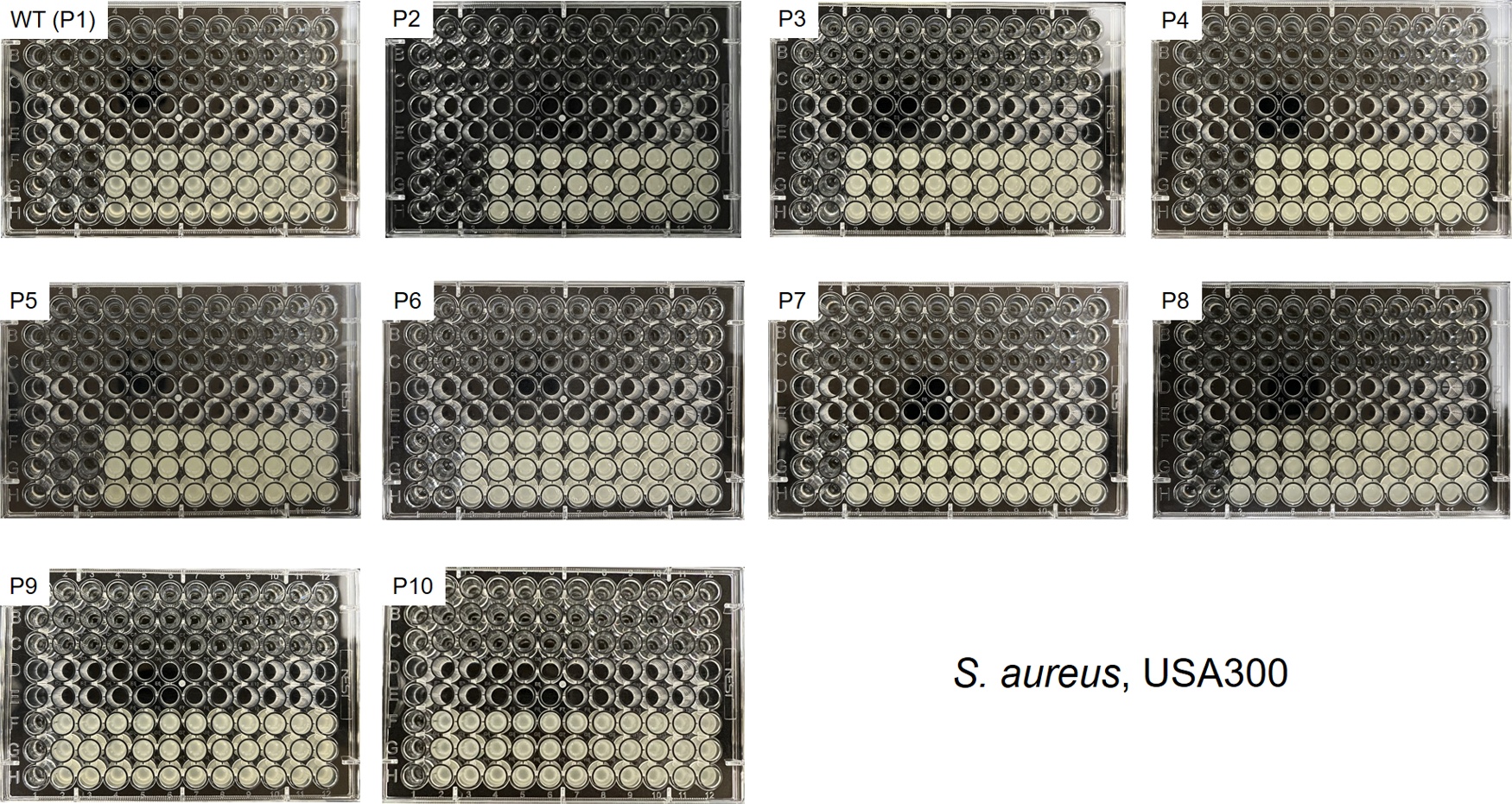
**

**Figure S18.** Evolution of ROS resistance in *S. aureus*, USA300. Photographs of the 96-well plates containing different passages (PN indicates passage N) of bacteria after cocultivation with H_2_O_2_ (Concentration decreasing 23 times in half from left to right, top to bottom) at 37 ^o^C for 24 h. The H_2_O_2_ concentration within the clear wells directly neighboring the turbid wells was recorded as the MIC. The treated bacteria that survived in these turbid wells with the highest H_2_O_2_ concentration were collected as the succeeding bacterial passage, and then subjected to a rechallenge employing an analogous methodology until P10 was reached.

**
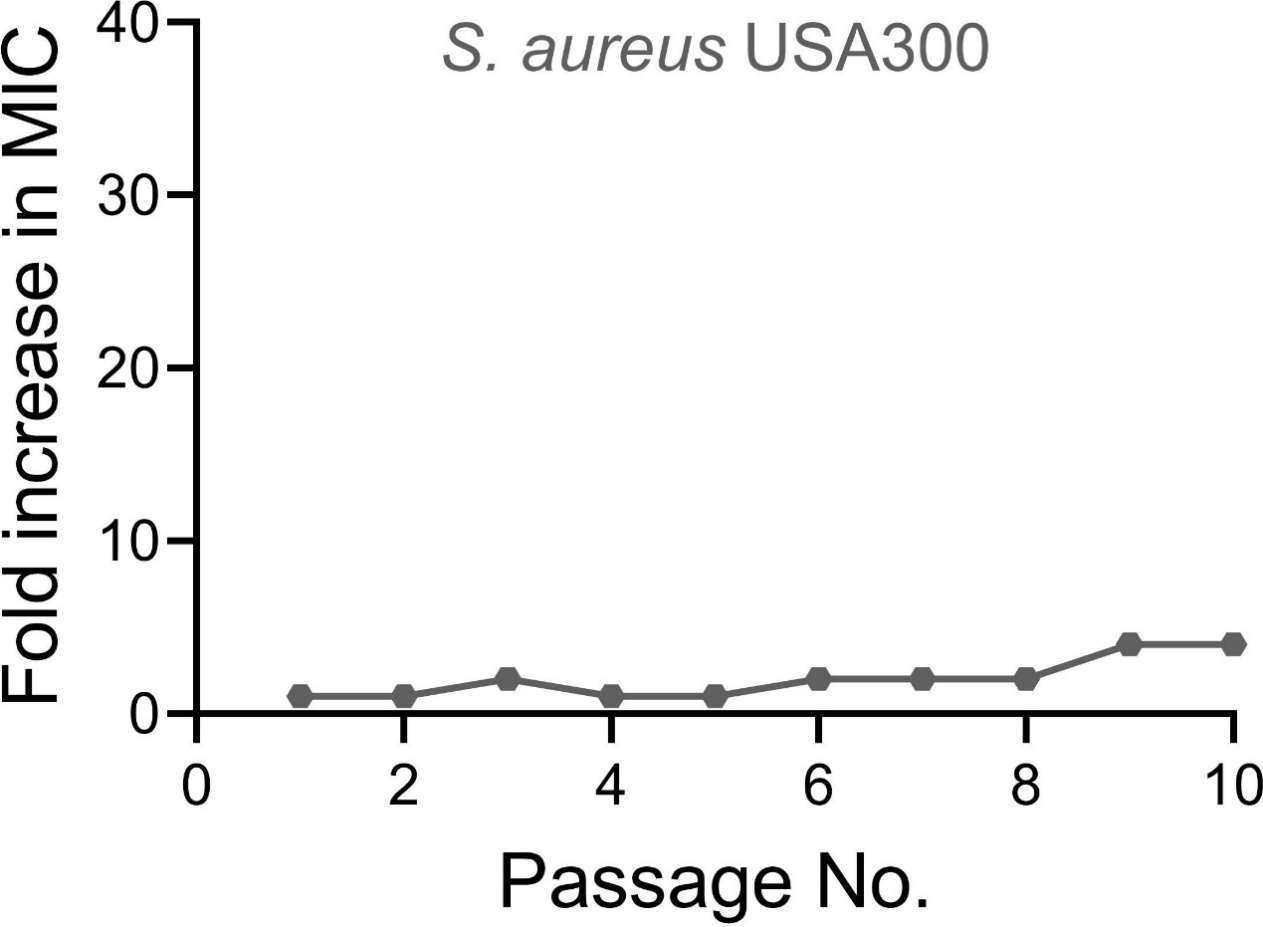
**

**Figure S19.** The development of ROS resistance in Gram-positive bacteria *S. aureus* USA300 is depicted in response to H_2_O_2_. This is represented by a fold increase in MIC versus the passage number. All experiments were performed in triplicate, with a sample size of three (n=3).
